# Association of Leisure Time Physical Activity Types and Risks of All-Cause, Cardiovascular, and Cancer Mortality Among Older Adults

**DOI:** 10.1001/jamanetworkopen.2022.28510

**Published:** 2022-08-24

**Authors:** Eleanor L. Watts, Charles E. Matthews, Joshua R. Freeman, Jessica S. Gorzelitz, Hyokyoung G. Hong, Linda M. Liao, Kathleen M. McClain, Pedro F. Saint-Maurice, Eric J. Shiroma, Steven C. Moore

**Affiliations:** 1 Division of Cancer Epidemiology and Genetics, National Cancer Institute, Rockville, Maryland; 2 Laboratory of Epidemiology and Population Science, National Institute on Aging, Bethesda, Maryland

## Abstract

**Importance:**

Higher amounts of physical activity are associated with increased longevity. However, whether different leisure time physical activity types are differentially associated with mortality risk is not established.

**Objectives:**

To examine whether participation in equivalent amounts of physical activity (7.5 to <15 metabolic equivalent of task [MET] hours per week) through different activity types is associated with mortality risk and to investigate the shape of the dose-response association.

**Design, Setting, and Participants:**

Participants in this cohort were respondents from the National Institutes of Health–AARP Diet and Health Study who completed the follow-up questionnaire between 2004 and 2005. This questionnaire collected data on weekly durations of different types of physical activities. Mortality was ascertained through December 31, 2019.

**Exposures:**

MET hours per week spent participating in the following activities: running, cycling, swimming, other aerobic exercise, racquet sports, golf, and walking for exercise.

**Main Outcomes and Measures:**

All-cause, cardiovascular, and cancer mortality. Separate multivariable-adjusted Cox proportional hazards regression models were fitted to estimate hazard ratios (HRs) and 95% CIs of mortality for each of the 7 types of leisure time physical activities, as well as the sum of these activities.

**Results:**

A total of 272 550 participants (157 415 men [58%]; mean [SD] age at baseline, 70.5 [5.4] years [range, 59-82 years]) provided information on types of leisure time activity, and 118 153 (43%) died during a mean (SD) follow-up of 12.4 (3.9) years. In comparison with those who did not participate in each activity, 7.5 to less than 15 MET hours per week of racquet sports (HR, 0.84; 95% CI, 0.75-0.93) and running (HR, 0.85; 95% CI, 0.78-0.92) were associated with the greatest relative risk reductions for all-cause mortality, followed by walking for exercise (HR, 0.91; 95% CI, 0.89-0.93), other aerobic activity (HR, 0.93; 95% CI, 0.90-0.95), golf (HR, 0.93; 95% CI, 0.90-0.97), swimming (HR, 0.95; 95% CI, 0.92-0.98), and cycling (HR, 0.97; 95% CI, 0.95-0.99). Each activity showed a curvilinear dose-response association with mortality risk; low MET hours per week of physical activity for any given activity type were associated with a large reduction in mortality risk, with diminishing returns for each increment in activity thereafter. Associations were similar for cardiovascular and cancer mortality.

**Conclusions and Relevance:**

This cohort study of older individuals found differences between different types of leisure time activities and mortality risk, but there were significant associations between participating in 7.5 to less than 15 MET hours per week of any activity and mortality risk.

## Key Points

**Question:**

Are different types of leisure time physical activity differentially associated with mortality risks among older adults?

**Findings:**

This cohort study of 272 550 older adults found that participation in 7.5 to less than 15 metabolic equivalent hours per week of running, cycling, swimming, other aerobic exercise, racquet sports, golf, and walking for exercise was associated with lower mortality risks compared with nonparticipants, although there were differences between risk estimates.

**Meaning:**

This study suggests that being physically active through participation in any type of leisure time activity is associated with lower mortality risks for older adults.

## Teaser

This cohort study uses data from the National Institutes of Health–AARP Diet and Health Study to examine whether participation in equivalent amounts of physical activity (7.5 to <15 metabolic equivalent hours/week) through different activity types is associated with mortality risk among older US adults.

## Introduction

People who participate in higher amounts of physical activity have a lower risk of mortality. A pooled prospective analysis including more than 660 000 participants estimated that achieving the recommended range of physical activity levels (7.5-15 metabolic equivalent of task [MET] hours per week) was associated with a 31% lower mortality risk in comparison with participants who did not achieve these activity levels.^1,2^

Despite the strong evidence of the beneficial role of physical activity in longevity, less is known about how engaging in the same amount of different leisure time activity types (such as running and cycling) are associated with mortality risks and whether some activities are associated with a greater benefit than others. Different associations with mortality risk among activity types could provide clues about variation in physiological adaptations conferring longevity; for instance, elite running is associated with increased early diastolic filling in comparison with swimmers,^3^ whereas swimmers have higher pulmonary function than other athletes.^4^

Previous prospective studies examining different types of activities were based on younger populations^5,6,7^ but were underpowered to assess dose-response associations, and those associations may not be generalizable to older adults.^2,8^ In addition, some studies have reported potential harms from very high levels of physical activity associated with some endurance sports.^9,10^ It is plausible that the potential harms from very high levels of physical activity may be greater for older adults. Participation in events, such as marathons, is increasing, and participants’ age is trending older.^11,12^ We aimed to compare mortality risk estimates for participating in comparable levels of different types of leisure time physical activities and to investigate the shape of the dose-response associations.

## Methods

### Data Source

The National Institutes of Health (NIH)–AARP Diet and Health study is a prospective cohort study designed to evaluate associations between diet and cancer. Details of the study protocol and assessment are available elsewhere.^13^ Between 1995 and 1996, 3.5 million baseline questionnaires were mailed to current AARP members aged 50 to 69 years who resided in California, Florida, Pennsylvania, New Jersey, North Carolina, and Louisiana or 2 metropolitan areas (Atlanta, Georgia, and Detroit, Michigan). A total of 567 169 questionnaires were returned. Between 2004 and 2005, follow-up questionnaires were mailed to the remaining cohort members.^14^ This follow-up questionnaire was completed by 313 363 participants. The NIH-AARP Diet and Health Study was approved by the Special Studies Institutional Review Board of the National Cancer Institute, and all participants gave written informed consent by completing and returning the questionnaire. Results were reported according to the Strengthening the Reporting of Observational Studies in Epidemiology (STROBE) reporting guideline for cohort studies.^15^

Our analytical data set included 272 550 participants who completed the follow-up questionnaire. We excluded baseline and follow-up proxy respondents (n = 18 493), respondents with missing or extreme body mass indexes (BMIs; calculated as weight in kilograms divided by height in meters squared) (<15 or >60; n = 3859), those who were unable to walk (n = 7287), and those missing more than 3 answers for the 7 leisure time physical activities examined (n = 11 174) (eFigure 1 in the Supplement). The characteristics of respondents who returned the baseline questionnaire were broadly similar to those who also returned the follow-up questionnaire (eTable 1 in the Supplement).

### Exposure Assessment

The follow-up questionnaire asked respondents to self-report mean time spent per week during the past year doing the following: (1) jogging or running; (2) cycling (including riding a stationary bike); (3) swimming laps; (4) other aerobic exercise (eg, aerobics class, using exercise machines); (5) playing tennis, squash, or racquetball; (6) playing golf; and (7) walking for exercise. Metabolic equivalent of task values were assigned to each activity and multiplied by the reported durations to estimate mean MET hours per week (eTable 2 in the Supplement).^16^

Respondents also self-reported height, weight, smoking status, depression, and trouble with physical activity, as well as other forms and durations of physical activity in the follow-up questionnaire. Sex, race and ethnicity, educational level, and alcohol consumption were assessed using the baseline questionnaire. History of diabetes, heart problems, stroke, emphysema, and cancer were determined using the combined responses to both the baseline and follow-up questionnaires. For further information on covariate assessment and selection, see the eMethods in the Supplement.

When BMI data were missing from the follow-up questionnaire, values were substituted from data collected at baseline (n = 26 772). Where participants had answered at least 3 questions for the 7 types of physical activities examined, missing answers for each respective activity were recoded to 0 (n = 39 093) under the assumption that nonresponse indicated that they did not do that activity.

### Participant Follow-up

Participants were followed up via record linkage to the US Postal Service National Change of Address database, through processing of undeliverable mail, address change services, and direct contact with participants. Determinations of vital status and causes of death were made through linkage with the National Death Index. Cancer mortality was defined as the primary cause of death only. Follow-up time was calculated from the date of return of the follow-up questionnaire until date of death or the date of censoring (December 31, 2019), whichever occurred first.

### Statistical Analysis

Hazard ratios (HRs) and 95% CIs of mortality were estimated using Cox proportional hazards regression models. We first examined the association of leisure time physical activity with all-cause, cardiovascular, and cancer mortality to confirm that the association in our analysis of older adults was similar to that reported by a prior pooled analysis.^1^ We refer specifically to categories given by the Physical Activity Guidelines for Americans, with the recommended level being 7.5 to less than 15 MET hours per week.^2^ We estimated the HRs for achieving 7.5 to less than 15 MET hours per week for each activity type vs no participation in the activity to allow the comparison of the strength of the association for activities of differing intensities at approximately similar volumes of energy expenditure. Finally, we assessed the association of different levels of each physical activity type with all-cause, cardiovascular, and cancer mortality. Participation in activities was categorized as none (nonparticipant), 0.1 to less than 7.5 (moderately active), 7.5 to less than 15 (active), 15 to less than 22.5 (highly active), and 22.5 or greater (very highly active) MET hours per week.

Our analyses adjusted for age; sex; racial and ethnic group; educational level; smoking status; BMI; alcohol consumption; marriage status; trouble with physical activity; history of stroke; history of myocardial infarction, angina, or coronary artery disease; history of emphysema; history of diabetes; ever received a diagnosis of cancer; total MET hours per week from non–leisure time activities; sedentary time; weight lifting; and total MET hours per week from leisure time activities (excluding the activity of interest) (eMethods in the Supplement). To understand the association of specific adjustments with outcomes, we added potential confounders and/or mediators sequentially to models in sensitivity analyses and compared results for each distinct adjustment. Adjustment categories were chosen a priori based on data availability and to maximize participant counts in each category.

We used E-values to assess the minimum strength of association that an unmeasured confounder would need with both exposure and outcome to explain away observed associations.^17,18^ To investigate the increased type I error rate over the 21 statistical tests (7 different activities and 3 outcomes), we also calculated the false-discovery rate for these analyses (*q* value threshold of 0.01).^19^

To test whether associations of each activity type with mortality risks were meaningfully different from one another, heterogeneity was assessed using χ^2^ tests. The proportional hazards assumption was assessed using log-log plots for each activity (eFigure 2 in the Supplement).

Subgroup analyses for participants who achieved 7.5 to less than 15 MET hours per week vs nonparticipants for the association of each activity type with all-cause mortality risk were examined using the following categories: follow-up time, age, sex, BMI, educational level, self-reported racial and ethnic group, smoking, trouble with physical activity, history of diabetes, history of heart problems, history of cancer, history of depression, and mean MET hours per week from the other activities. Subgroup definitions and tests for heterogeneity are described in greater detail in the eMethods in the Supplement. We additionally examined whether achieving 7.5 to less than 15 MET hours per week through a combination of any 2 types of physical activities was associated with all-cause mortality risk (in comparison with those who did not participate in either activity) and associations for any amount of participation in each activity in comparison with nonparticipants.

All analyses were performed using SAS, version 9.4 (SAS Institute Inc); Stata, version 17.0 (StataCorp LLC); and R, version 3.2.3 (R Group for Statistical Computing). The figures were created in R using the Jasper,^20^ survminer, and ggplot2 packages. All tests of significance were 2-sided, and *P* < .01 was considered statistically significant as a conservative estimate owing to the number of statistical tests.

## Results

A total of 272 550 participants (157 415 men [58%]; mean [SD] age at baseline, 70.5 [5.4] years [range, 59-82 years]) provided information on types of leisure time activity. After a mean (SD) follow-up of 12.4 (3.9) years, 118 153 participants (43%) died, including 38 300 from cardiovascular disease and 32 366 from cancer. Walking for exercise was the most common type of activity (78% of participants), followed by other aerobic exercise (30%), cycling (25%), golf (14%), swimming (10%), running (7%), and racquet sports (4%).

Respondents who achieved 7.5 to less than 15 MET hours per week for running, aerobic exercise, racquet sports, and walking were younger on average, while swimmers were older (Table 1). Those who were active through any of the activities also had a lower BMI (except swimmers) and had higher levels of participation in other activities (except golfers). Each physical activity type was weakly positively correlated with the other activity types (ranging from *r* = 0.01 for racquet sports and walking to *r* = 0.22 for aerobic exercise and cycling) (eTable 3 in the Supplement).

**Table 1.** Characteristics of the Participants in the National Institutes of Health–AARP Study by Leisure Time Physical Activity Types

Characteristic	Running, MET h/wk		Cycling, MET h/wk		Swimming, MET h/wk		Aerobic, MET h/wk		Golf, MET h/wk		Racquet sports, MET h/wk		Walking for exercise, MET h/wk	
	0	7.5 to <15	0	7.5 to <15	0	7.5 to <15	0	7.5 to <15	0	7.5 to <15	0	7.5 to <15	0	7.5 to <15
No.	254 307	1759	205 580	22 585	245 373	7629	189 703	13 692	233 234	6411	262 404	1017	60 940	47 856
Age, mean (SD), y	70.6 (5.3)	68.6 (5.2)	70.5 (5.4)	70.5 (5.3)	70.5 (5.4)	70.7 (5.3)	70.5 (5.4)	70.4 (5.3)	70.5 (5.4)	70.5 (5.4)	70.5 (5.4)	70.1 (5.3)	70.5 (5.4)	70.4 (5.3)
BMI, mean (SD)	27.1 (4.9)	25.3 (3.5)	27.1 (5.0)	26.7 (4.4)	27.0 (4.8)	27.0 (4.7)	27.2 (4.9)	26.4 (4.5)	27.1 (5.0)	26.7 (3.9)	27.1 (4.9)	25.5 (3.5)	27.6 (5.3)	26.5 (4.4)
Frequency, mean, h/wk	0	1.5	0	1.2	0	1.2	0	1.5	0	2.5	0	1.5	0	2.5
MET from other activities, mean (SD), h/wk	59.7 (51.3)	77.6 (63.6)	56.0 (48.2)	66.9 (49.5)	59.4 (51.7)	74.3 (59.8)	54.7 (48.2)	57.9 (42.8)	58.0 (52.8)	54.8 (44.5)	60.9 (54.1)	63.5 (51.7)	48.7 (44.8)	52.3 (39.6)
Sex, %														
Male	56	82	55	65	58	57	60	49	53	76	57	76	58	58
Female	44	18	45	35	43	24	47	24	42	42	42	43	40	51
Self-reported race and ethnicity, %														
Asian	1	2	1	1	1	1	1	1	1	1	1	2	1	1
Hispanic	2	2	2	2	2	2	2	1	2	1	2	1	1	1
American Indian/Alaska Native	0.2	0.2	0.2	0.2	0.2	0.3	0.2	0.2	0.2	0.2	0.2	0.1	0.2	0.2
Non-Hispanic Black	3	4	3	3	3	1	3	3	4	1	3	2	3	2
Non-Hispanic White	93	90	93	93	93	95	93	93	92	96	93	93	93	94
Pacific Islander	0.1	0.1	0.1	0.0	0.1	0.0	0.1	0.1	0.1	0.0	0.1	0.1	0.1	0.1
Missing	1	1	1	1	1	1	1	1	1	1	1	1	1	1
Educational level, %														
Up to 11 y	4	2	4	3	4	2	4	2	4	2	4	1	4	3
12 y or completed high school	17	8	18	15	17	12	18	13	18	13	17	8	19	15
Post–high school	9	7	9	9	9	8	10	8	10	8	10	4	10	9
Some college	23	18	23	22	23	21	23	23	23	24	23	14	23	23
College graduate and postgraduate	44	63	44	49	44	54	42	52	43	52	44	72	42	49
Missing	2	2	2	2	2	2	2	2	2	2	2	1	2	2
Smoking, %														
Never	37	42	37	38	37	38	36	39	38	31	37	42	33	39
Former														
<20 Cigarettes/d	26	28	26	28	26	28	25	29	26	29	26	27	24	27
≥20 Cigarettes/d	22	19	22	22	22	20	23	21	21	26	22	20	24	21
Current														
<20 Cigarettes/d	3	2	3	2	3	3	3	2	3	2	3	2	4	2
≥20 Cigarettes/d	3	1	3	1	3	2	3	1	3	2	3	1	5	2
Unknown	9	8	9	9	9	9	9	9	9	9	9	9	10	9
Alcohol, g/d, %														
Nondrinker	21	15	22	18	21	14	22	17	23	11	21	10	22	19
<10	50	50	50	51	50	51	49	54	51	50	50	50	48	51
10-19	13	16	12	15	13	16	12	14	12	18	13	20	12	14
≥20	16	19	16	16	15	18	16	14	14	21	15	20	17	15
Unknown	0.4	0.2	0.4	0.4	0.4	0.3	0.4	0.4	0.4	0.4	0.4	0.5	0.5	0.4
Marriage status, %														
Married or living as married	69	81	68	74	69	70	70	68	67	82	69	79	69	71
Not married or living as married	30	19	31	26	30	29	30	32	33	17	30	21	30	28
Unknown	0.6	0.5	0.6	0.5	0.6	0.7	0.6	0.5	0.6	0.5	0.6	0.5	0.6	0.5
Trouble with physical activity, %														
None	41	58	42	42	42	43	41	44	41	48	42	51	39	45
Slight amount	29	25	28	30	29	29	28	31	29	30	29	30	27	30
Moderate amount	14	7	14	14	14	14	14	12	15	12	14	9	15	13
Quite a bit	7	2	7	6	7	6	7	5	7	4	7	2	9	5
An enormous amount	1	0.4	1	1	1	0.9	1	0.7	1	0.3	1	0.5	2	0.7
Missing	7	6	7	7	7	7	7	7	7	7	7	7	8	7
Ever had diabetes, %														
No	76	84	76	78	76	78	75	80	76	78	76	83	76	78
Yes	16	9	16	14	16	13	17	12	16	13	16	9	16	14
Unknown	8	7	8	8	8	8	8	8	8	9	8	8	8	8
Ever had myocardial infarction, angina, or coronary artery disease, %														
No	73	79	73	71	73	74	73	74	73	72	73	77	74	73
Yes	21	15	20	24	21	19	21	20	21	22	21	17	20	21
Unknown	6	6	6	5	6	6	6	6	6	6	6	6	6	6
Ever had a stroke, %														
No	89	92	89	89	89	89	88	90	89	89	89	90	88	90
Yes	4	2	4	4	4	3	4	3	4	3	4	2	4	3
Unknown	7	7	7	7	7	8	8	7	7	7	7	8	8	7
Ever had emphysema, %														
No	83	89	83	85	83	84	83	85	83	85	83	87	81	85
Yes	8	3	9	7	8	7	9	7	9	6	8	4	10	7
Unknown	8	8	8	8	8	9	9	8	8	9	8	9	9	8
Ever had cancer, %														
No	62	63	62	62	62	62	62	61	62	61	62	59	62	62
Yes	28	25	28	27	28	29	28	28	28	29	28	29	28	28
Unknown	10	12	10	10	10	9	10	11	10	10	10	12	10	10

Abbreviations: BMI, body mass index (calculated as weight in kilograms divided by height in meters squared); MET, metabolic equivalent of task.

**Figure 1.**
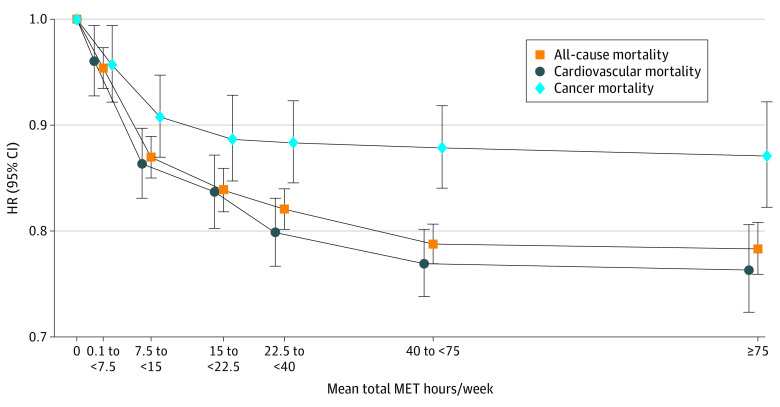
Associations of Mean Total Sum Metabolic Equivalent of Task (MET) Hours per Week of the 7 Activities With All-Cause, Cardiovascular, and Cancer Mortality Categories are based on the Physical Activity Guidelines for Americans.^2^ Hazard ratios (HRs) were adjusted for age; sex; racial and ethnic group; educational level; smoking status; body mass index; alcohol consumption; marriage status; trouble with physical activity; history of stroke; history of myocardial infarction, angina, or coronary artery disease; history of diabetes; ever received a diagnosis of cancer; total MET hours per week from nonleisure time activities; sedentary time; and weight training frequency. The data points indicate the HRs, and vertical lines indicate 95% CIs. The line joinings the data points are to illustrate the shape of the dose-response association. The position of the categories along the x-axis was based on the reported median MET hours per week.

The shape of the association for overall leisure time physical activity with all-cause, cardiovascular, and cancer mortality risks were curvilinear, with smaller magnitudes of associations observed for cancer mortality (Figure 1). Individuals who were moderately active through a combination of the 7 activities (0.1 to <7.5 MET hours per week) had a 5% lower risk of all-cause mortality than those who did not participate in these activities (HR, 0.95; 95% CI, 0.94-0.97), while those who were active (7.5 to <15 MET hours per week) had a 13% lower risk (HR, 0.87; 95% CI, 0.85-0.89). Highly active participants (≥15 MET hours per week) also had lower risks of mortality, although higher total MET hours per week were associated with relatively smaller reductions in mortality risk.

Participation in 7.5 to less than 15 MET hours per week for each of the individual types of physical activities was associated with lower risks of mortality, but the magnitude of the associations with all-cause mortality and cardiovascular mortality differed by activity type (both *P* < .001 for heterogeneity). In comparison with nonparticipation in each activity, participation in 7.5 to less than 15 MET hours per week for racquet sports (HR, 0.84; 95% CI, 0.75-0.93) and running (HR, 0.85; 95% CI, 0.78-0.92) was associated with the greatest risk reductions in all-cause mortality, followed by walking for exercise (HR, 0.91; 95% CI, 0.89-0.93), other aerobic activity (HR, 0.93; 95% CI, 0.90-0.95), golf (HR, 0.93; 95% CI, 0.90-0.97), swimming (HR, 0.95; 95% CI, 0.92-0.98), and cycling (HR, 0.97; 95% CI, 0.95-0.99) (Table 2). For cardiovascular mortality, playing racquet sports was associated with the greatest reduction in mortality (HR, 0.73; 95% CI, 0.59-0.89); for cancer mortality, running was associated with the largest risk reduction (HR, 0.81; 95% CI, 0.69-0.95). The estimated false discovery rate was 0.0% given the magnitude of associations observed and our *P* < .01 threshold. The E-values indicated that large amounts of unmeasured confounding would be required to explain observed associations with mortality, particularly for the risk estimates with the largest magnitudes (eTable 4 in the Supplement). For example, the E-value of 1.49 for 7.5 to less than 15 MET hours per week of running indicates that an unmeasured confounder would need to be associated with a greater than 1.49-fold increase in risk for both the exposure and all-cause mortality. In sensitivity analyses, adjusting for health-related variables and other types of physical activity had the greatest association with outcomes among statistical adjustments, with modest attenuation of associations when these factors were included in the model (eTable 5 in the Supplement).

**Table 2.** Associations of Leisure Time Physical Activity Types With All-Cause, Cardiovascular, and Cancer Mortality in National Institutes of Health–AARP Diet and Health Study Participants

Activity	All-cause mortality			Cardiovascular mortality			Cancer mortality		
	Deaths	HR (95% CI)^a^	*P* value	Deaths	HR (95% CI)^a^	*P* value	Deaths	HR (95% CI)^a^	*P* value
Running, MET h/wk									
0	112 325	1 [Reference]	<.001	36 457	1 [Reference]	.27	30 637	1 [Reference]	.009
7.5 to <15	524	0.85 (0.78-0.92)		176	0.92 (0.79-1.07)		153	0.81 (0.69-0.95)	
Cycling, MET h/wk									
0	91 052	1 [Reference]	.008	29 284	1 [Reference]	.99	25 099	1 [Reference]	.004
7.5 to <15	9345	0.97 (0.95-0.99)		3164	1.00 (0.96-1.04)		2509	0.94 (0.90-0.98)	
Swimming, MET h/wk									
0	107 366	1 [Reference]	.005	34 749	1 [Reference]	.67	29 313	1 [Reference]	.08
7.5 to <15	3119	0.95 (0.92-0.98)		1046	0.99 (0.93-1.05)		855	0.94 (0.88-1.01)	
Aerobic exercise, MET h/wk									
0	86 023	1 [Reference]	<.001	27 922	1 [Reference]	.08	23 422	1 [Reference]	.001
7.5 to <15	5169	0.93 (0.90-0.95)		1719	0.96 (0.91-1.01)		1404	0.91 (0.86-0.97)	
Racquet sports, MET h/wk									
0	114 759	1 [Reference]	.001	37 274	1 [Reference]	.002	31 293	1 [Reference]	.88
7.5 to <15	347	0.84 (0.75-0.93)		94	0.73 (0.59-0.89)		121	1.01 (0.85-1.21)	
Golf, MET h/wk									
0	102 175	1 [Reference]	.001	33 283	1 [Reference]	.008	27 418	1 [Reference]	.95
7.5 to <15	2658	0.93 (0.90-0.97)		845	0.91 (0.85-0.98)		801	1.00 (0.93-1.08)	
Walking for exercise, MET h/wk									
0	28 506	1 [Reference]	<.001	9197	1 [Reference]	<.001	7736	1 [Reference]	.05
7.5 to <15	19 325	0.91 (0.89-0.93)		6201	0.89 (0.86-0.92)		5527	0.97 (0.93-1.00)	
Test for heterogeneity			<.001			<.001			.14

Abbreviations: HR, hazard ratio; MET, metabolic equivalent of task.

^a^ Adjusted for age; sex; racial and ethnic group; educational level; smoking status; body mass index; alcohol consumption; marriage status; trouble with physical activity; history of stroke; history of myocardial infarction, angina, or coronary artery disease; history of diabetes; ever received a diagnosis of cancer; total MET hours per week from nonleisure time activities; sedentary time; weight training frequency; and total MET hours per week from other leisure time activities (excluding the activity of interest). The heterogeneity between HRs for each activity type and mortality risk was assessed using the χ^2^ test.

**Figure 2.**
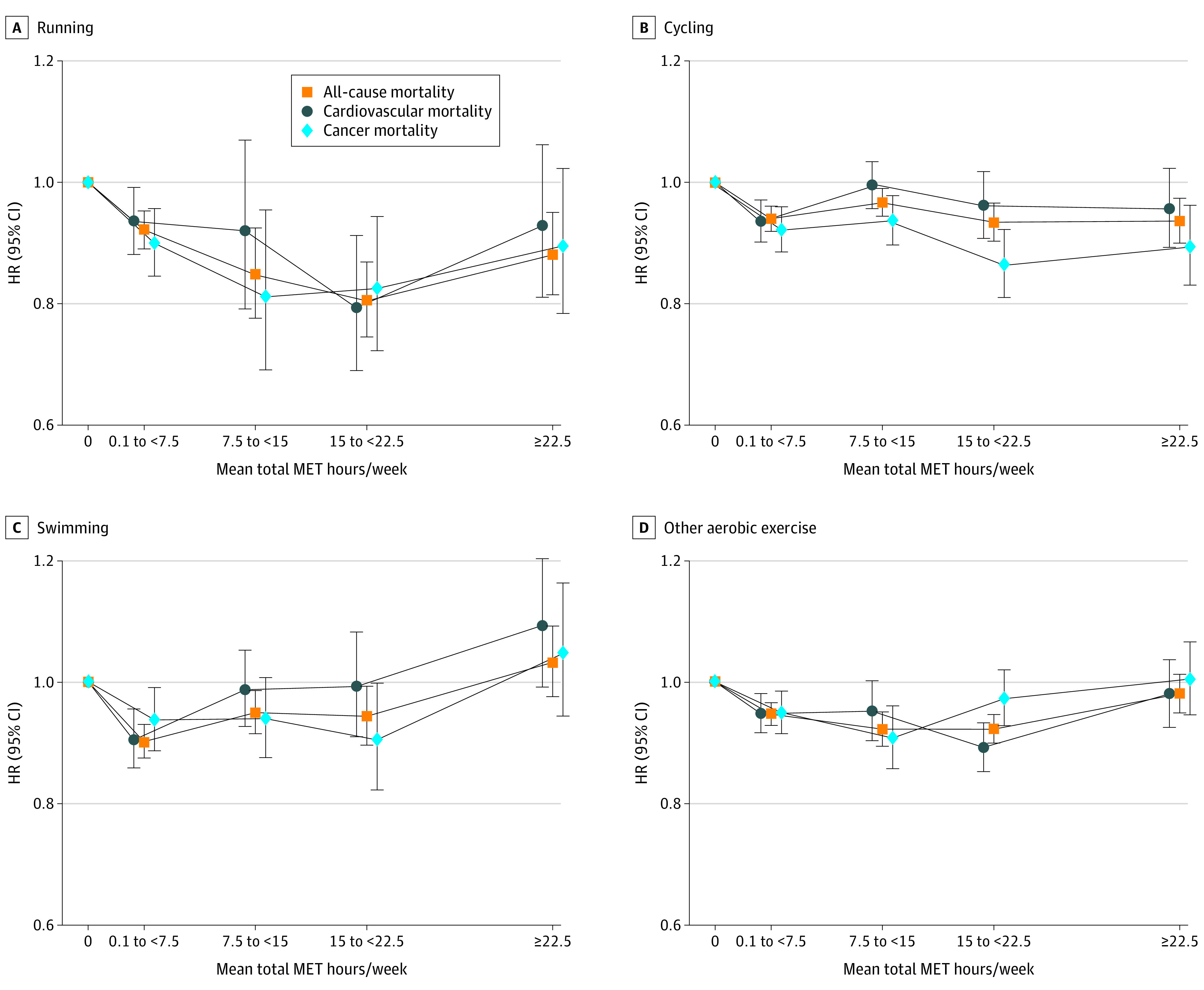
Associations of Mean Metabolic Equivalent of Task (MET) Hours per Week of Running, Cycling, Swimming, and Other Aerobic Activities With All-Cause, Cardiovascular, and Cancer Mortality Hazard ratios (HRs) were adjusted for age; sex; racial and ethnic group; educational level; smoking status; body mass index; alcohol consumption; marriage status; trouble with physical activity; history of stroke; history of myocardial infarction, angina, or coronary artery disease; history of diabetes; ever received a diagnosis of cancer; total MET hours per week from nonleisure time activities; sedentary time; weight training frequency; and total MET hours per week from other leisure time activities (excluding the activity of interest). The data points indicate the HRs, and vertical lines indicate 95% CIs. The lines joining the data points are to illustrate the shape of the dose-response association.

**Figure 3.**
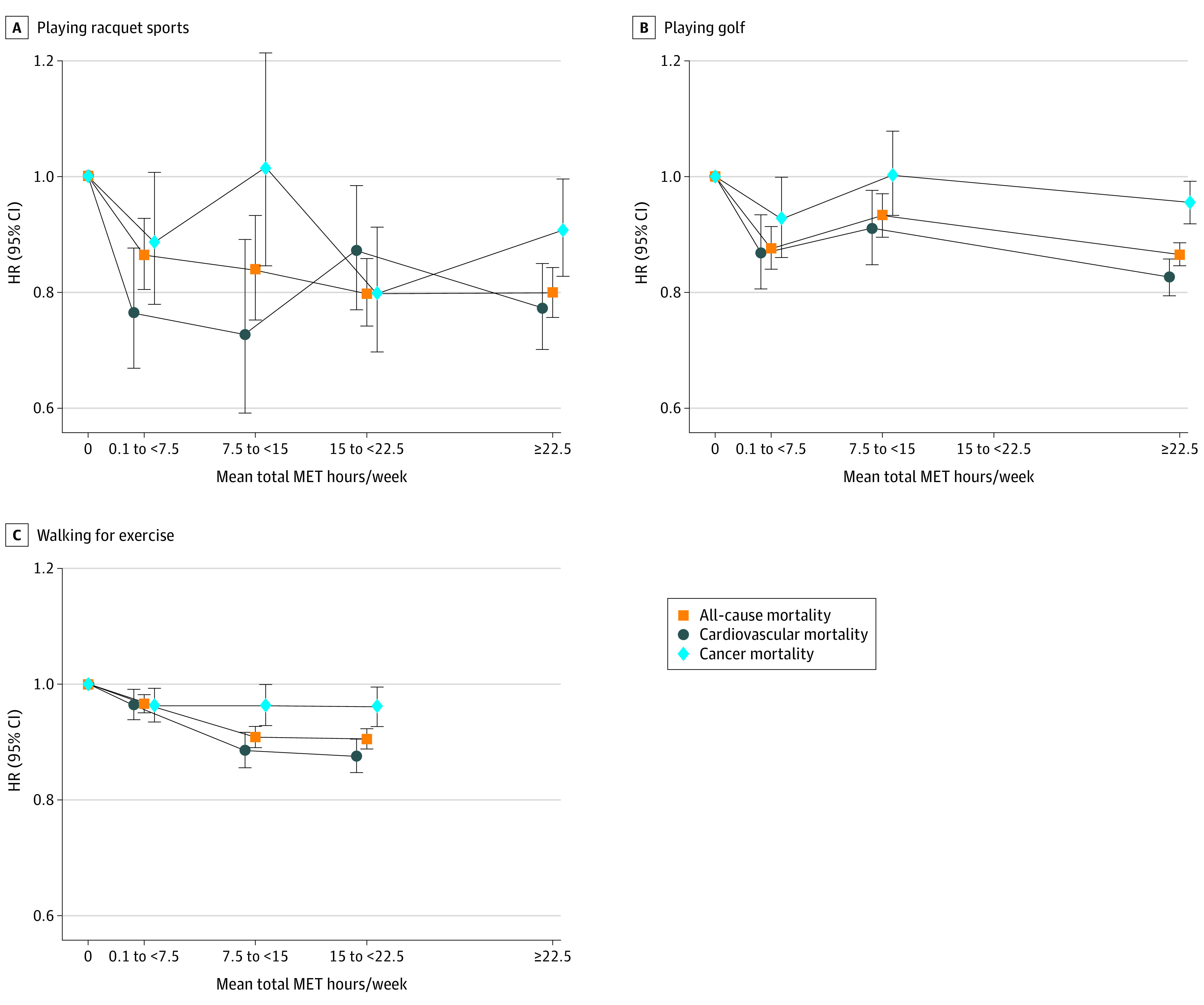
Associations of Mean Metabolic Equivalent of Task (MET) Hours per Week of Racquet Sports, Golf, and Walking With All-Cause, Cardiovascular, and Cancer Mortality Hazard ratios (HRs) were adjusted for age; sex; racial and ethnic group; educational level; smoking status; body mass index; alcohol consumption; marriage status; trouble with physical activity; history of stroke; history of myocardial infarction, angina, or coronary artery disease; history of diabetes; ever received a diagnosis of cancer; total MET hours per week from nonleisure time activities; sedentary time; weight training frequency; and total MET hours per week from other leisure time activities (excluding the activity of interest). The data points indicate the HRs, and vertical lines indicate 95% CIs. The lines joining the data points are to illustrate the shape of the dose-response association.

The shapes of the associations of each activity type with mortality risks were broadly similar to the shape of the association of overall activity with mortality (Figure 2 and Figure 3). Individuals who were moderately active to active through each activity (0.1 to <15 MET hours per week) had lower mortality risks, including for cardiovascular and cancer mortality, than nonparticipants. Highly active individuals (>15 MET hours per week through each activity) generally had lower mortality risks than those who only just met the recommendations but with diminishing returns as activity levels increase. For runners, swimmers, and those performing aerobic exercise, there was evidence of higher mortality risks for individuals who reported very high levels of these activities (≥22.5 MET hours per week) when compared with those who reported 15 to less than 22.5 MET hours per week. Risk estimates and 95% CIs are available in eTable 6 in the Supplement.

### Subgroup Analyses

Our subgroup analyses were exploratory; their purpose was to examine how robust our estimates were across different contexts and possible confounders for exploration in future studies. There was evidence of heterogeneity in the associations of activity types and all-cause mortality risk by sex; associations with running were null for women (HR, 1.09; 95% CI, 0.88-1.34) and inverse for men (HR, 0.80; 95% CI, 0.73-0.88; *P* = .01 for heterogeneity), whereas associations for walking were stronger for women (HR, 0.88; 95% CI, 0.86-0.91) than men (HR, 0.93; 95% CI, 0.90-0.95; *P* = .004 for heterogeneity) (eFigure 3 in the Supplement). Swimming 7.5 to less than 15 MET hours per week for individuals with a BMI of less than 25 was associated with a reduction in mortality risk (HR, 0.87; 95% CI, 0.82-0.92), whereas associations were null for those with higher BMIs (BMI, 25-29.9: HR, 0.98; 95% CI, 0.93-1.04; and BMI, ≥30: HR, 1.07; 95% CI, 0.99-1.15; *P* < .001 for heterogeneity). Playing golf was associated with lower mortality risks for those who were not college graduates (up to high school: HR, 0.88; 95% CI, 0.80-0.97; high school and some college: HR, 0.87; 95% CI, 0.82-0.94), while associations for college graduates were null (HR, 0.99; 95% CI, 0.94-1.05; *P* = .01 for heterogeneity). The magnitude of the associations for cycling, aerobic exercise, and walking were stronger for participants with less than 5 years of follow-up than those with 5 years or more of follow-up (eFigure 3 in the Supplement). When we stratified by level of participation in other leisure time activities, the associations for any one activity were consistently strongest among those who did little other activity. This was particularly true for cycling, aerobic exercise, and walking (*P* < .001 for heterogeneity) (eTable 7 in the Supplement).

Where respondents achieved 7.5 to less than 15 MET hours per week through a combination of 2 different activities, risks were not materially different than those reported in our primary analysis (eTable 8 in the Supplement). When we compared risks for any participation in each activity vs no participation, racquet sports had the largest magnitude of association with all-cause mortality (HR, 0.82; 95% CI, 0.79-0.85) (eTable 9 in the Supplement).

## Discussion

Our comprehensive analysis of more than 270 000 older adults with a mean of 12 years of follow-up demonstrates the benefits associated with participating in any of these leisure time physical activity types for reducing mortality risk, including cardiovascular and cancer mortality, among older populations. Although all types of activities were associated with lower risks of mortality, participants who achieved 7.5 to less than 15 MET hours per week for racquet sports and running had the lowest all-cause mortality risk in comparison with those who participated in other activities. Each activity showed a curvilinear dose-response association with mortality risk; low levels of physical activity were associated with a large reduction in mortality risk, with diminishing returns for each increment in activity thereafter.

The larger reduction in risk of mortality associated with running and racquet sports than for other activity types for older adults may be associated with the specific physiological demands and adaptations that occur with these sports.^3,4^ These activities both require synchronized action from many muscles for correct form, and racquet sports also require hand-eye coordination and intermittent bursts of very high intensity, which may additionally improve physical functioning.^21,22^ This combination of types of action is in line with the Physical Activity Guidelines for Americans, which recommended that older adults engage in multicomponent physical activity, including balance training as well as aerobic and muscle-strengthening activities.^2^

Our findings that all types of activities are associated with greater longevity support the evidence that leisure time physical activity can reduce the risk of mortality.^1,2^ Although we found differences in associations between activities, participation in any of the activities was associated with lower mortality in comparison with those who did not participate in each activity, including moderate-intensity activities. Previous prospective studies based on younger adults have generally reported protective associations for most types of leisure time activities with mortality risks, although results were not consistently statistically significant.^5,6,7^ Our analysis expands on previous studies by using an older population with a larger sample size and long duration of follow-up. We found a universal benefit to older individuals associated with physical activity. Our study had greater power to investigate the shape of the associations for each activity.

Most individual activity types had associations similar in shape to that for all activities combined. However, individuals who reported very high levels of participation in higher-intensity activities (ie, running, swimming, and aerobic activity) had slightly higher mortality risk in comparison with those who reported more moderate levels. Other prospective studies have also reported similar associations for participants who report high levels of physical activity,^9,10^ putatively owing to adverse cardiovascular remodeling in response to the chronic stress of endurance activities.^23^ However, this population is relatively older, and the 95% CIs for those with very high levels of each activity were large. The observed higher risks for this group may also be associated with adjustment for factors that are on the causal pathway^24,25^ (such as comorbid conditions), overestimation of physical activities, or reporting other types of physical activities that may not have been interpreted as being independent (such as indoor cyclists). These limitations reduce the reliability of our risk estimates for individuals who report high levels of physical activity, while the benefits associated with lower levels of physical activity in comparison with less active individuals are likely more robust.

### Strengths and Limitations

This study has some strengths, including its large population and long duration of follow-up, which maximizes statistical power for evaluating the individual activities and enables subgroup analyses across a range of variables. This population comprises older adults, for whom the shape of the association of physical activity with mortality is less well characterized. We focus on discrete types of structured physical activity, which are recalled with higher reliability than light, unstructured activities (such as housework).^26^ Data were collected and compared across a wide range of activity types.

This study also has some limitations. Our study is observational; although we controlled for relevant measured confounders, we cannot fully exclude the possibility that residual confounding by smoking, socioeconomic status, or other activities may have biased effect estimates. To minimize this possibility, we included adjustments for factors such as BMI and other health factors that may be on the causal pathway, so our risk estimates may be conservative. The follow-up questionnaire also did not examine lower-intensity activities, such as yoga or stretching. The large sample size also meant that very modest associations were statistically significant. We also did not include muscle-strengthening activity as a primary exposure measure, as the recommendation for strengthening activity is distinct from recommendations for aerobic activity. Respondents were asked to recall their mean physical activity participation within the past 12 months; therefore, information on longer-term physical activity, earlier-life physical activity, or physical activity during follow-up is not known. A previous study reported moderate correlations between self-reported physical activity type measured at baseline and a second measurement of physical activity type 2 years later among the same participants (intraclass correlation coefficient, 0.79 for racquet sports and 0.38 for walking and hiking).^27^ Recall error and change in behavior may attenuate risk estimates and distort the shape of the dose-response curve.^26^ The Physical Activity Guidelines for Americans recommend that older adults determine their level of effort for physical activity relative to their level of fitness.^2^ However, the follow-up questionnaire did not collect data on perceived intensity or other metrics, such as speed. Future research, including the use of objective measures of physical activity, such as accelerometers, may help to improve the precision of these measures. Finally, this population was predominantly White, older, and with high socioeconomic status; therefore, risk estimates might not be generalizable to other populations.

## Conclusions

This large cohort study suggests that achieving 7.5 to less than 15 MET hours per week for any type of moderate to vigorous leisure time activity is associated with a lower risk of mortality among an older population. Our dose-response analysis also indicates that even small amounts of physical activity may be associated with reduced mortality for individuals who do not engage in leisure time physical activity, but additional benefits associated with physical activity may be smaller for individuals who are highly active. Finally, although we report differences between the associations of activity types with mortality, all types of activity were associated with lower mortality risk; therefore, finding an activity that older, inactive individuals enjoy (and so may sustain) is likely of a greater benefit than choosing a particular activity based on the differences between risk estimates reported.

## Notes

Supplement.
eMethods.
**eTable 1.** Characteristics of Participants at Baseline With No Follow-up Questionnaire Data vs Those With Data **eTable 2.** MET Values for the 7 Leisure Time Physical Activity Types **eTable 3.** Spearman Rank Correlation Coefficients of Physical Activity Disciplines **eTable 4.** E-values for Associations of Different Physical Activity Types With All-Cause, Cardiovascular and Cancer Mortality in NIH-AARP Diet and Health Study Participants (7.5 to <15 MET hrs/wk vs None) **eTable 5.** Associations of Activity Types With All-Cause Mortality Following Sequential Adjustment for Covariates in NIH-AARP Diet and Health Study Participants **eTable 6.** Categorical Associations of Leisure Time Physical Activity Types With All-Cause, Cardiovascular and Cancer Mortality in NIH-AARP Diet and Health Study Participants **eTable 7.** Associations of Achieving 7.5 to <15 MET Hours per Week Through Each Activity Type With All-Cause Mortality by Average MET Hours per Week From Other Activities **eTable 8.** Associations of Two Activity Types With All-Cause Mortality in NIH-AARP Diet and Health Study Participants **eTable 9.** Associations of Each Activity With All-Cause, Cardiovascular and Cancer Mortality for NIH-AARP Diet and Health Study Participants (Any vs None) **eFigure 1.** Participant Inclusion Flow Chart **eFigure 2.** Log-Log Plots to Assess Proportional Hazards Assumption With All-Cause Mortality **eFigure 3.** Associations of Activity Types With All-Cause Mortality by Subgroups
eReferences.
