# Supplementary material for: Association of Leisure Time Physical Activity Types and Risks of All-Cause, Cardiovascular, and Cancer Mortality Among Older Adults
Source: JAMA Netw Open. 2022 Aug 24;5(8):e2228510. doi: 10.1001/jamanetworkopen.2022.28510 (PMC9403775; doi:10.1001/jamanetworkopen.2022.28510)
Supplement: Supplement. — eMethods. eTable 1. Characteristics of Participants at Baseline With No Follow-up Questionnaire Data vs Those With Data eTable 2. MET Values for the 7 Leisure Time Physical Activity Types eTable 3. Spearman Rank Correlation Coefficients of Physical Activity Disciplines eTable 4. E-values for Associations of Different Physical Activity Types With All-Cause, Cardiovascular and Cancer Mortality in NIH-AARP Diet and Health Study Participants (7.5 to <15 MET hrs/wk vs None) eTable 5. Associations of Activity Types With All-Cause Mortality Following Sequential Adjustment for Covariates in NIH-AARP Diet and Health Study Participants eTable 6. Categorical Associations of Leisure Time Physical Activity Types With All-Cause, Cardiovascular and Cancer Mortality in NIH-AARP Diet and Health Study Participants eTable 7. Associations of Achieving 7.5 to <15 MET Hours per Week Through Each Activity Type With All-Cause Mortality by Average MET Hours per Week From Other Activities eTable 8. Associations of Two Activity Types With All-Cause Mortality in NIH-AARP Diet and Health Study Participants eTable 9. Associations of Each Activity With All-Cause, Cardiovascular and Cancer Mortality for NIH-AARP Diet and Health Study Participants (Any vs None) eFigure 1. Participant Inclusion Flow Chart eFigure 2. Log-Log Plots to Assess Proportional Hazards Assumption With All-Cause Mortality eFigure 3. Associations of Activity Types With All-Cause Mortality by Subgroups eReferences. [file jamanetwopen-e2228510-s001.pdf]

Supplementary Online Content

Watts EL, Matthews CE, Freeman JR, et al. Association of leisure time physical activity types and risks of all-cause, cardiovascular, and cancer mortality among older adults. *JAMA Netw Open.* 2022;5(8):e2228510. doi:10.1001/jamanetworkopen.2022.28510

eMethods.
eTable 1. Characteristics of Participants at Baseline With No Follow-up Questionnaire Data vs Those With Data
eTable 2. MET Values for the 7 Leisure Time Physical Activity Types
eTable 3. Spearman Rank Correlation Coefficients of Physical Activity Disciplines
eTable 4. E-values for Associations of Different Physical Activity Types With All-Cause, Cardiovascular and Cancer Mortality in NIH-AARP Diet and Health Study Participants (7.5 to <15 MET hrs/wk vs None)
eTable 5. Associations of Activity Types With All-Cause Mortality Following Sequential Adjustment for Covariates in NIH-AARP Diet and Health Study Participants
eTable 6. Categorical Associations of Leisure Time Physical Activity Types With All-Cause, Cardiovascular and Cancer Mortality in NIH-AARP Diet and Health Study Participants
eTable 7. Associations of Achieving 7.5 to <15 MET Hours per Week Through Each Activity Type With All-Cause Mortality by Average MET Hours per Week From Other Activities
eTable 8. Associations of Two Activity Types With All-Cause Mortality in NIH-AARP Diet and Health Study Participants
eTable 9. Associations of Each Activity With All-Cause, Cardiovascular and Cancer Mortality for NIH-AARP Diet and Health Study Participants (Any vs None)
eFigure 1. Participant Inclusion Flow Chart
eFigure 2. Log-Log Plots to Assess Proportional Hazards Assumption With All-Cause Mortality
eFigure 3. Associations of Activity Types With All-Cause Mortality by Subgroups
eReferences.

This supplementary material has been provided by the authors to give readers additional information about their work.

eMethods.

Exposure assessment

The follow-up questionnaire asked respondents to self-report average time spent per week over the last year: i) jogging or running, ii) cycling (including riding a stationary bike), iii) swimming laps, iv) playing golf, v) playing tennis, squash, or racquetball, vi) other aerobic exercise (e.g., aerobic class, exercise machines, etc.), vii) walking for exercise. There were 10 possible response options (none, 5 min, 15 min, 30 min, 1 hour, 1 hour 30 min, 2–3 hours, 4–6 hours, 7–10 hours, more than 10 hours). MET values were assigned to each activity and multiplied by the reported durations to estimate average MET hours per week (Supplementary Table 2)[1]. Where durations were a range of values, the midpoint duration of the range was assigned, where participants selected >10 hours, we assumed 12 hours of activity. Overall leisure time physical activity was calculated as the sum of the MET hours per week of the seven activities examined.

Respondents also self-reported: height, weight, smoking status (number of cigarettes per day current and historically), depression (diagnosed by doctor) and trouble with physical activity (none, slight, moderate, quite a bit, an enormous amount), and other forms and durations of physical activity in the follow-up questionnaire (light household chores, moderate to vigorous household chores, moderate outdoor chores, home repairs, caring for children, caring for another adult, and walking for other daily activities, weight training or lifting) and sedentary time (sitting watching television, video, or DVD, sitting or driving in a car, bus or train, other sitting (reading, knitting, using a computer)). Sex (male and female), race/ethnicity (American Indian or Alaskan Native, Asian, Hispanic, non-Hispanic Black, non-Hispanic White, Pacific Islander; categories defined by questionnaire), education level (<8 years, 8-11, completed high school, post-high school training excluding college, some college, college graduate, postgraduate) and alcohol consumption (g/day estimated based on beer, wine and liquor/mixed drink frequency and portion sizes over the last 12 months) were assessed using the baseline questionnaire. Doctor diagnosed history of diabetes, heart problems, stroke, emphysema, and cancer were determined using the self-reported combined responses to both the baseline and follow-up questionnaires (ever, never, or unknown). Co-morbidities for adjustments were chosen *a priori* based on the possibility that they may affect both physical activity levels at baseline and mortality risks[2-4].

When BMI data were missing from the follow-up questionnaire, values were substituted from data collected at baseline (n=26,772). Where participants had answered at least three questions for the seven types of physical activities examined, missing answers for each respective activity were recoded to 0 (n=39,093), under the assumption that nonresponse indicated that they did not do that activity.

© 2022 Watts EL et al. *JAMA Network Open*.

Adjustment covariates

Our primary analyses adjusted for age (continuous), sex (male, female), racial/ethnic group (Asian, Hispanic, non-Hispanic Black, non-Hispanic White, Pacific Islander or American Indian/Alaskan Native, and missing [1.0%]), education (less than 11 years, 12 years or completed high school, post-high school, some college, college and post graduate, and missing [2.2%]), smoking status (never, former light smoker (<20 cigarettes per day), former heavy smoker (20+ cigarettes per day), current light smoker (<20 cigarettes per day), current heavy smoker (20+ cigarettes per day), and missing [9.2%]), BMI (≤20, 20.0-22.4, 22.5-24.9, 25.0-27.4, 27.5-29.9, 30.0-31.9, 32.0-33.9, and ≥34 kg/m^2), alcohol consumption (non-drinker, <10, 10-19, 20+ g alcohol per day, and missing [0.4%]), marriage status (married or living as married, widowed/divorced/separated/never married, and missing [0.6%]), trouble with physical activity (none, slight amount, moderate amount, quite a bit, an enormous amount, and missing [7.2%]), history of stroke (yes, no, and missing [7.3%]), history of heart attack, angina, or coronary artery disease (yes, no, and missing [6.0%]), history of emphysema (yes, no, and missing [8.4%]), history of diabetes (yes, no, and missing [8.2%]), ever been diagnosed with cancer (yes, no, and missing [10.1%]), total MET hours per week from non-leisure time activities (light household chores, moderate to vigorous household chores, moderate outdoor chores, home repairs, caring for children, caring for another adult, and walking for other daily activities, fifths, missing [11.8%]), sedentary time (time spent watching television, sitting during travel, and other sitting, fifths, missing [16.3%]), weight lifting frequency (none, ≤1, >1 hour/week, missing [1.8%]), and total MET hours from the other leisure time activities (running, cycling, swimming, aerobic exercise, racquet sports, golf, walking for exercise, excluding the activity of interest, in fifths assigned the median value, continuous).

E-values

E-values were used to assess the minimum strength of association that an unmeasured confounder would need with both exposure and outcome to explain away observed associations[5, 6]. HRs were transformed using the formula:

$$RR \approx \frac{1 - 0.5^{\sqrt{HR}}}{1 - 0.5^{\sqrt{1/HR}}}$$

And the inverse of the RR was substituted into the equation[6]:

$$E - value = RR + \sqrt{RR \times (RR - 1)}$$

© 2022 Watts EL et al. *JAMA Network Open*.

If the upper confidence interval crossed 0, the interval bound was = 1, else the upper CI was substituted into the E-value formula.

Subgroup analyses

Subgroup analyses for the associations of meeting the physical activity recommendations vs nonparticipants through each activity type and all-cause mortality risk were examined using the following categories: follow-up time (<5; 5+ years), age (<65, 65-69, 70-74, 75+ years), sex (male, female), BMI (<25, 25-29.9, 30+ kg/m^2), education level (up to high school, high school-college, college graduate), self-reported racial group (Non-Hispanic Black, Non-Hispanic White), smoking (never, former, current), physical activity trouble (none, slight/moderate, quite a bit), history of diabetes (no, yes), history of heart problems (no, yes), history of cancer (no, yes), history of depression (no, yes), and average MET hours per week from the other six activities (< 7.5, 7.5+ MET hours/week). Heterogeneity in the associations for follow-up time was examined using in two different subgroups defined by follow-up period, using a $\chi2$ test for heterogeneity. Heterogeneity in the associations for the non-case dependent subgroups was assessed using a $\chi2$ interaction term.

© 2022 Watts EL et al. *JAMA Network Open*.

eTable 1. Characteristics of Participants at Baseline With no Follow-up Questionnaire Data vs Those With Data

		Baseline cohort, no Follow-up questionnaire (*N*=253,035)	Baseline cohort, with Follow-up questionnaire (*N*=313,363)	Percentage difference
Age (SD)		62.4 (5.4)	62.0 (5.3)	-0.8
BMI (SD)		27.3 (5.3)	26.9 (4.9)	-1.4
Sex	Male	61.6	58.6	-3.0
	Female	38.4	41.4	3.0
Self-reported race and ethnicity, %	Non-Hispanic White	89.8	92.5	2.7
	Non-Hispanic Black	4.6	3.3	-1.3
	Hispanic	2.2	1.6	-0.5
	Asian	1.2	1.2	0.0
	Pacific Islander	0.1	0.1	0.0
	Native American/Alaskan	0.4	0.2	-0.1
	Missing	1.7	1.1	-0.7
Education, %	Up to 11 years	8.9	4.4	-4.5
	12 years or completed high school	22.4	17.5	-5.0
	Post high school	10.3	9.4	-0.9
	Some college	23.4	22.7	-0.8
	College graduate and postgraduate	31.2	43.7	12.6
	Missing	3.8	2.4	-1.4
Smoking, %	Never	30.5	38.1	7.5
	Former smoker <20 p/day	27.0	28.5	1.5
	Former smoker 20+ p/day	23.1	20.6	-2.5
	Current smoker <20 p/day	9.4	6.4	-3.0
	Current smoker 20+ p/day	5.6	3.1	-2.5
	Unknown	4.5	3.4	-1.1
Alcohol (g alc p/day), %	Non-drinker	27.9	21.6	-6.3
	< 10	45.8	49.8	4.1
	10-19	10.4	12.5	2.1
	20 +	15.5	15.6	0.1
	Unknown	0.5	0.4	-0.1
Marriage status, %	Married/living as married	67.2	70.2	3.0
	Not married/living as married	31.8	29.2	-2.5
	Unknown	1.1	0.6	-0.5
Ever had diabetes, %	No	88.1	92.8	4.7
	Yes	11.9	7.2	-4.7
Ever had heart disease, %	No	83.2	88.0	4.8
	Yes	16.9	12.0	-4.9
Ever had a stroke, %	No	96.8	98.5	1.6
	Yes	3.2	1.5	-1.6
Ever had emphysema, %	No	95.9	98.2	2.3
	Yes	4.1	1.8	-2.3
Ever had cancer, %	No	84.2	87.9	3.7
	Yes	10.4	7.8	-2.6
	Unknown	5.4	4.3	-1.1

Abbreviations: SD=standard deviation

© 2022 Watts EL et al. *JAMA Network Open*.

eTable 2. MET Values for the 7 Leisure Time Physical Activity Types

Activity type	MET values
Running	7.0
Cycling	7.5
Swimming	8.3
Aerobic	7.3
Racquet sport	7.3
Golf	4.8
Walking for exercise	4.3

MET values based on the Compendium of Physical Activities[1]

Abbreviations: MET= metabolic equivalent of task.

© 2022 Watts EL et al. *JAMA Network Open*.

eTable 3. Spearman Rank Correlation Coefficients of Physical Activity Disciplines

	Running	Cycling	Swimming	Aerobic	Racquet sports	Golf	Walking for exercise
Running	1						
Cycling	0.11	1					
Swimming	0.07	0.13	1				
Aerobic	0.13	0.22	0.1	1			
Racquet sports	0.11	0.08	0.07	0.05	1		
Golf	0.05	0.07	0.05	0.03	0.09	1	
Walking for exercise	0.08	0.09	0.06	0.14	0.01	0.06	1

© 2022 Watts EL et al. *JAMA Network Open.*

eTable 4. E-values for Associations of Different Physical Activity Types With All-Cause, Cardiovascular and Cancer Mortality in NIH-AARP Diet and Health Study Participants (7.5 to <15 MET hrs/wk vs None)

	E-values (interval bound)		
	All-cause mortality	Cardiovascular mortality	Cancer mortality
Running	1.49 (1.31)	1.31 (1.00)	1.59 (1.23)
Cycling	1.17 (1.08)	1.01 (1.00)	1.26 (1.13)
Swimming	1.23 (1.12)	1.11 (1.00)	1.26 (1.00)
Aerobic exercise	1.29 (1.22)	1.22 (1.00)	1.33 (1.18)
Racquet sports	1.51 (1.28)	1.80 (1.39)	1.11 (1.00)
Golf	1.27 (1.17)	1.33 (1.15)	1.04 (1.00)
Walking for exercise	1.33 (1.29)	1.39 (1.32)	1.18 (1.00)

E-values are calculated based on the results displayed in Table 2

© 2022 Watts EL et al. *JAMA Network Open.*

eTable 5. Associations of Activity Types With All-Cause Mortality Following Sequential Adjustment for Covariates in NIH-AARP Diet and Health Study Participants

	Running			Cycling			Swimming			Aerobic	
Model iteration	HR (95% CI)	P		HR (95% CI)	P		HR (95% CI)	P		HR (95% CI)	P
1	0.66 (0.61, 0.72)	<0.0001		0.88 (0.86, 0.90)	<0.0001		0.86 (0.83, 0.90)	<0.0001		0.80 (0.78, 0.82)	<0.0001
2	0.68 (0.63, 0.74)	<0.0001		0.89 (0.87, 0.91)	<0.0001		0.87 (0.83, 0.90)	<0.0001		0.81 (0.79, 0.84)	<0.0001
3	0.68 (0.63, 0.74)	<0.0001		0.89 (0.87, 0.91)	<0.0001		0.86 (0.83, 0.90)	<0.0001		0.81 (0.79, 0.84)	<0.0001
4	0.70 (0.64, 0.76)	<0.0001		0.90 (0.88, 0.92)	<0.0001		0.88 (0.85, 0.91)	<0.0001		0.83 (0.81, 0.86)	<0.0001
5	0.74 (0.68, 0.80)	<0.0001		0.93 (0.91, 0.95)	<0.0001		0.90 (0.87, 0.93)	<0.0001		0.86 (0.84, 0.88)	<0.0001
6	0.80 (0.73, 0.87)	<0.0001		0.92 (0.90, 0.94)	<0.0001		0.91 (0.88, 0.94)	<0.0001		0.88 (0.85, 0.90)	<0.0001
7	0.84 (0.77, 0.92)	0.0001		0.96 (0.94, 0.98)	0.0004		0.94 (0.90, 0.97)	0.0002		0.92 (0.90, 0.95)	<0.0001
8	0.85 (0.78, 0.92)	0.0001		0.97 (0.95, 0.99)	0.008		0.95 (0.92, 0.98)	0.005		0.93 (0.90, 0.95)	<0.0001

HRs represent mortality risks for achieving 7.5-15 MET hrs/wk through each activity in comparison with those who did not participate in each activity.
Model 1=adjusted for age (continuous), sex (male, female).
Model 2 = Model 1 + adjusted for BMI (≤20, 20.0-22.4, 22.5-24.9, 25.0-27.4, 27.5-29.9, 30.0-31.9, 32.0-33.9, and ≥34 kg/m^2).
Model 3 = Model 2 + adjusted for racial/ethnic group (non-Hispanic white, non-Hispanic black, Hispanic, Asian, Pacific Islander or American Indian/Alaskan Native, and missing [1.0%]).
Model 4 = Model 3 + adjusted for education (less than 11 years, 12 years or completed high school, post-high school, some college, college and postgraduate, and missing [2.2%]), and marriage status (married or living as married, widowed/divorced/separated/never married, and missing [0.6%]).
Model 5 = Model 4 + adjusted for smoking status (never, former light smoker (<20 cigarettes per day), former heavy smoker (20+ cigarettes per day), current light smoker (<20 cigarettes per day), current heavy smoker (20+ cigarettes per day), and missing [9.2%]), and alcohol consumption (non-drinker, <10, 10-19, 20+ g alcohol per day, and missing [0.4%]).
Model 6 = Model 5 + adjusted for trouble with physical activity (none, slight amount, moderate amount, quite a bit, an enormous amount, and missing [7.2%]), history of stroke (yes, no, and missing [7.3%]), history of heart attack, angina, or coronary artery disease (yes, no, and missing [6.0%]), history of emphysema (yes, no, and missing [8.4%]), history of diabetes (yes, no, and missing [8.2%]), ever been diagnosed with cancer (yes, no, and missing [10.1%]).
Model 7 = Model 6 + adjusted for total MET hours per week from non-leisure time activities (light household chores, moderate to vigorous household chores, moderate outdoor chores, home repairs, caring for children, caring for another adult, and walking for other daily activities, fifths, missing [11.8%]), sedentary time (fifths, missing [16.3%]), weight training frequency (none, ≤1, >1 hour/week, missing [1.8%]).
Model 8 = Model 7 + adjusted for total MET hours per week from other activities (running, cycling, swimming, aerobic exercise, racquet sports, golf, walking for exercise, excluding the activity of interest, fifths assigned the median value, continuous).
Abbreviations: CI=confidence interval; HR=hazard ratio; MET= metabolic equivalent of task; NIH-AARP=National Institutes of Health-AARP.

© 2022 Watts EL et al. *JAMA Network Open.*

eTable 5. Associations of Activity Types With All-Cause Mortality Following Sequential Adjustment for Covariates in NIH-AARP Diet and Health Study Participants (continued)

	Racquet sports			Golf			Walking	
Model iteration	HR (95% CI)	P		HR (95% CI)	P		HR (95% CI)	P
1	0.68 (0.62, 0.76)	<0.0001		0.86 (0.83, 0.90)	<0.0001		0.80 (0.78, 0.81)	<0.0001
2	0.71 (0.63, 0.78)	<0.0001		0.88 (0.84, 0.91)	<0.0001		0.82 (0.80, 0.83)	<0.0001
3	0.71 (0.64, 0.79)	<0.0001		0.88 (0.84, 0.91)	<0.0001		0.82 (0.80, 0.83)	<0.0001
4	0.74 (0.66, 0.82)	<0.0001		0.89 (0.86, 0.93)	<0.0001		0.83 (0.81, 0.84)	<0.0001
5	0.77 (0.70, 0.86)	<0.0001		0.89 (0.86, 0.93)	<0.0001		0.87 (0.85, 0.89)	<0.0001
6	0.82 (0.74, 0.91)	0.0003		0.93 (0.89, 0.96)	0.0002		0.89 (0.87, 0.90)	<0.0001
7	0.83 (0.75, 0.92)	0.0005		0.93 (0.90, 0.97)	0.0004		0.91 (0.89, 0.93)	<0.0001
8	0.84 (0.75, 0.93)	0.001		0.93 (0.90, 0.97)	0.0005		0.91 (0.89, 0.93)	<0.0001

HRs represent mortality risks for achieving 7.5-15 MET hrs/wk through each activity in comparison with those who did not participate in each activity.
Model 1 = adjusted for age (continuous), sex (male, female).
Model 2 = Model 1 + adjusted for BMI (≤20, 20.0-22.4, 22.5-24.9, 25.0-27.4, 27.5-29.9, 30.0-31.9, 32.0-33.9, and ≥34 kg/m^2).
Model 3 = Model 2 + adjusted for racial/ethnic group (non-Hispanic white, non-Hispanic black, Hispanic, Asian, Pacific Islander or American Indian/Alaskan Native, and missing [1.0%]).
Model 4 = Model 3 + adjusted for education (less than 11 years, 12 years or completed high school, post-high school, some college, college and postgraduate, and missing [2.2%]), and marriage status (married or living as married, widowed/divorced/separated/never married, and missing [0.6%]).
Model 5 = Model 4 + adjusted for smoking status (never, former light smoker (<20 cigarettes per day), former heavy smoker (20+ cigarettes per day), current light smoker (<20 cigarettes per day), current heavy smoker (20+ cigarettes per day), and missing [9.2%]), and alcohol consumption (non-drinker, <10, 10-19, 20+ g alcohol per day, and missing [0.4%]).
Model 6 = Model 5 + adjusted for trouble with physical activity (none, slight amount, moderate amount, quite a bit, an enormous amount, and missing [7.2%]), history of stroke (yes, no, and missing [7.3%]), history of heart attack, angina, or coronary artery disease (yes, no, and missing [6.0%]), history of emphysema (yes, no, and missing [8.4%]), history of diabetes (yes, no, and missing [8.2%]), ever been diagnosed with cancer (yes, no, and missing [10.1%]).
Model 7 = Model 6 + adjusted for total MET hours per week from non-leisure time activities (light household chores, moderate to vigorous household chores, moderate outdoor chores, home repairs, caring for children, caring for another adult, and walking for other daily activities, fifths, missing [11.8%]), sedentary time (fifths, missing [16.3%]), weight training frequency (none, ≤1, >1 hour/week, missing [1.8%]).
Model 8 = Model 7 + adjusted for total MET hours per week from other activities (running, cycling, swimming, aerobic exercise, racquet sports, golf, walking for exercise, excluding the activity of interest, fifths assigned the median value, continuous).
Abbreviations: CI=confidence interval; HR=hazard ratio; MET= metabolic equivalent of task; NIH-AARP=National Institutes of Health-AARP.

© 2022 Watts EL et al. *JAMA Network Open.*

eTable 6. Categorical Associations of Leisure Time Physical Activity Types With All-Cause, Cardiovascular and Cancer Mortality in NIH-AARP Diet and Health Study Participants

		All-cause mortality	Cardiovascular mortality	Cancer mortality
	MET hrs/week	HR (95% CI)	HR (95% CI)	HR (95% CI)
Running	None	1 (ref)	1 (ref)	1 (ref)
	0.1-<7.5	0.92 (0.89, 0.95)	0.93 (0.88, 0.99)	0.90 (0.85, 0.95)
	7.5-<15	0.85 (0.78, 0.92)	0.92 (0.79, 1.07)	0.81 (0.69, 0.95)
	15-<22.5	0.80 (0.75, 0.87)	0.79 (0.69, 0.91)	0.83 (0.72, 0.94)
	22.5+	0.88 (0.82, 0.95)	0.93 (0.81, 1.06)	0.90 (0.79, 1.02)
Cycling	None	1 (ref)	1 (ref)	1 (ref)
	0.1-<7.5	0.94 (0.92, 0.96)	0.94 (0.90, 0.97)	0.92 (0.89, 0.96)
	7.5-<15	0.97 (0.95, 0.99)	0.99 (0.96, 1.03)	0.94 (0.90, 0.98)
	15-<22.5	0.93 (0.90, 0.96)	0.96 (0.91, 1.02)	0.86 (0.81, 0.92)
	22.5+	0.94 (0.90, 0.97)	0.96 (0.89, 1.02)	0.89 (0.83, 0.96)
Swimming	None	1 (ref)	1 (ref)	1 (ref)
	0.1-<7.5	0.90 (0.88, 0.93)	0.90 (0.86, 0.95)	0.94 (0.89, 0.99)
	7.5-<15	0.95 (0.92, 0.98)	0.99 (0.93, 1.05)	0.94 (0.88, 1.01)
	15-<22.5	0.94 (0.90, 0.99)	0.99 (0.91, 1.08)	0.91 (0.82, 1.00)
	22.5+	1.03 (0.98, 1.09)	1.09 (0.99, 1.20)	1.05 (0.94, 1.16)
Aerobic	None	1 (ref)	1 (ref)	1 (ref)
	0.1-<7.5	0.95 (0.93, 0.96)	0.95 (0.92, 0.98)	0.95 (0.92, 0.98)
	7.5-<15	0.92 (0.90, 0.95)	0.95 (0.90, 1.00)	0.91 (0.86, 0.96)
	15-<22.5	0.92 (0.90, 0.94)	0.89 (0.85, 0.93)	0.97 (0.93, 1.02)
	22.5+	0.98 (0.95, 1.01)	0.98 (0.93, 1.04)	1.00 (0.95, 1.06)
Racquet sport	None	1 (ref)	1 (ref)	1 (ref)
	0.1-<7.5	0.86 (0.81, 0.93)	0.77 (0.67, 0.87)	0.89 (0.78, 1.01)
	7.5-<15	0.84 (0.75, 0.93)	0.73 (0.59, 0.89)	1.01 (0.85, 1.21)
	15-<22.5	0.80 (0.74, 0.86)	0.87 (0.77, 0.98)	0.80 (0.70, 0.91)
	22.5+	0.80 (0.76, 0.84)	0.77 (0.70, 0.85)	0.91 (0.83, 0.99)
Golf	None	1 (ref)	1 (ref)	1 (ref)
	0.1-<7.5	0.88 (0.84, 0.91)	0.87 (0.81, 0.93)	0.93 (0.86, 1.00)
	7.5-<15	0.93 (0.90, 0.97)	0.91 (0.85, 0.97)	1.00 (0.93, 1.08)
	15-<22.5	-	-	-
	22.5+	0.87 (0.85, 0.88)	0.83 (0.80, 0.86)	0.95 (0.92, 0.99)
Walking for exercise	None	1 (ref)	1 (ref)	1 (ref)
	0.1-<7.5	0.97 (0.95, 0.98)	0.96 (0.94, 0.99)	0.96 (0.94, 0.99)
	7.5-<15	0.91 (0.89, 0.92)	0.89 (0.86, 0.91)	0.96 (0.93, 1.00)
	15-<22.5	0.90 (0.89, 0.92)	0.88 (0.85, 0.90)	0.96 (0.93, 0.99)
	22.5+	-	-	-

HRs were adjusted for age, sex, racial/ethnic group, education, smoking status, BMI, alcohol consumption, marriage status, trouble with physical, history of stroke, history of heart attack, angina, or coronary artery disease, history of diabetes, ever been diagnosed with cancer, total MET hours per week from non-leisure time activities, sedentary time, weight training frequency and total MET hours per week from other leisure time activities (excluding the activity of interest).

Abbreviations: CI=confidence interval; HR=hazard ratio; MET=metabolic equivalent of task; NIH-AARP=National Institutes of Health-AARP.

© 2022 Watts EL et al. *JAMA Network Open*.

eTable 7. Associations of Achieving 7.5 to <15 MET Hours per Week Through Each Activity Type With All-Cause Mortality by Average MET Hours per Week From Other Activities

Activity type	MET hours/week from other leisure time activities[a]	Deaths	HR (95% CI)	P_{het}
Running	Low	46,614	0.68 (0.55, 0.84)	
	High	66,235	0.89 (0.81, 0.97)	0.03
Cycling	Low	46,279	0.92 (0.89, 0.96)	
	High	54,118	1.00 (0.97, 1.02)	0.002
Swimming	Low	46,724	0.90 (0.84, 0.97)	
	High	63,761	0.97 (0.93, 1.01)	0.09
Aerobic exercise	Low	45,593	0.85 (0.81, 0.90)	
	High	45,599	0.97 (0.94, 1.01)	0.0001
Racquet sports	Low	47,122	0.77 (0.61, 0.97)	
	High	67,984	0.87 (0.77, 0.98)	0.36
Golf	Low	47,514	0.89 (0.84, 0.95)	
	High	57,319	0.96 (0.92, 1.01)	0.07
Walking for exercise	Low	29,603	0.88 (0.86, 0.91)	
	High	18,228	0.96 (0.93, 0.99)	0.0001

[a]Low=<7.5; high=7.5 + MET hours per week.
HRs for the associations of achieving 7.5-<15 MET hours per week through each activity in comparison with those who did not participate in each activity with all-cause mortality, stratified by average MET hours per week from the other six activities. HRs were adjusted for age, sex, racial/ethnic group, education, smoking status, BMI, alcohol consumption, marriage status, trouble with physical, history of stroke, history of heart attack, angina, or coronary artery disease, history of diabetes, ever been diagnosed with cancer, total MET hours per week from non-leisure time activities, sedentary time, weight training frequency and total MET hours per week from other leisure time activities (excluding the activity of interest).
Abbreviations: BMI=body mass index; CI=confidence interval; HR=hazard ratio; MET=metabolic equivalent of task.

© 2022 Watts EL et al. *JAMA Network Open.*

eTable 8. Associations of Two Activity Types With All-Cause Mortality in NIH-AARP Diet and Health Study Participants

	Running			Cycling			Swimming		
	Deaths/total	HR (95% CI)	P	Deaths/total	HR (95% CI)	P	Deaths/total	HR (95% CI)	P
Running				625/1904	0.91(0.84,0.98)	0.02	252/773	0.90(0.80,1.02)	0.11
Cycling	625/1904	0.91(0.84,0.98)	0.02				958/2493	0.94(0.88,1.00)	0.06
Swimming	252/773	0.90(0.80,1.02)	0.11	958/2493	0.94(0.88,1.00)	0.06			
Aerobic	675/2178	0.86(0.80,0.93)	0.0001	3139/7712	0.94(0.91,0.98)	0.003	836/2310	0.86(0.80,0.92)	<0.0001
Racquet sport	102/314	0.88(0.72,1.06)	0.18	221/628	0.97(0.85,1.10)	0.61	101/307	0.88(0.72,1.06)	0.18
Golf	148/431	0.92(0.79,1.09)	0.34	498/1342	0.85(0.77,0.92)	0.0002	224/589	0.90(0.79,1.02)	0.10
Walking for exercise	1345/3993	0.90(0.85,0.95)	0.0002	6426/15663	0.91(0.88,0.94)	<0.0001	2432/6263	0.87(0.83,0.91)	<0.0001

HRs represent mortality risks for those achieving 7.5-<15 MET hrs/wk through a combination of two activities in comparison with those who did not participate in either activity.

HRs were adjusted for age, sex, racial/ethnic group, education, smoking status, BMI, alcohol consumption, marriage status, trouble with physical, history of stroke, history of heart attack, angina, or coronary artery disease, history of diabetes, ever been diagnosed with cancer, total MET hours per week from non-leisure time activities, sedentary time, weight training frequency and total MET hours per week from other leisure time activities (excluding the two activities of interest).

Abbreviations: CI=confidence interval; HR=hazard ratio; MET= metabolic equivalent of task; NIH-AARP=National Institutes of Health-AARP.

© 2022 Watts EL et al. *JAMA Network Open.*

eTable 8. Associations of Two Activity Types With All-Cause Mortality in NIH-AARP Diet and Health Study Participants (continued)

	Aerobic				Racquet sport		
	Deaths/total	HR (95% CI)	P		Deaths/total	HR (95% CI)	P
Running	675/2178	0.86(0.80,0.93)	0.0001		102/314	0.88(0.72,1.06)	0.18
Cycling	3139/7712	0.94(0.91,0.98)	0.003		221/628	0.97(0.85,1.10)	0.61
Swimming	836/2310	0.86(0.80,0.92)	<0.0001		101/307	0.88(0.72,1.06)	0.18
Aerobic					187/579	0.88(0.77,1.02)	0.09
Racquet sport	187/579	0.88(0.77,1.02)	0.09				
Golf	444/1219	0.88(0.80,0.97)	0.01		68/195	0.99(0.78,1.26)	0.95
Walking for exercise	6178/15431	0.89(0.86,0.91)	<0.0001		336/1021	0.81(0.73,0.90)	0.0001

HRs represent mortality risks for those achieving 7.5-<15 MET hrs/wk through a combination of two activities in comparison with those who did not participate in either activity. HRs were adjusted for age, sex, racial/ethnic group, education, smoking status, BMI, alcohol consumption, marriage status, trouble with physical, history of stroke, history of heart attack, angina, or coronary artery disease, history of diabetes, ever been diagnosed with cancer, total MET hours per week from non-leisure time activities, sedentary time, weight training frequency and total MET hours per week from other leisure time activities (excluding the two activities of interest).
Abbreviations: CI=confidence interval; HR=hazard ratio; MET= metabolic equivalent of task; NIH-AARP=National Institutes of Health-AARP.

© 2022 Watts EL et al. *JAMA Network Open.*

eTable 8. Associations of Two Activity Types With All-Cause Mortality in NIH-AARP Diet and Health Study Participants (continued)

	Golf				Walking for exercise		
	Deaths/total	HR (95% CI)	P		Deaths/total	HR (95% CI)	P
Running	148/431	0.92(0.79,1.09)	0.34		1345/3993	0.90(0.85,0.95)	0.0002
Cycling	498/1342	0.85(0.77,0.92)	0.0002		6426/15663	0.91(0.88,0.94)	<0.0001
Swimming	224/589	0.90(0.79,1.02)	0.10		2432/6263	0.87(0.83,0.91)	<0.0001
Aerobic	444/1219	0.88(0.80,0.97)	0.007		6178/15431	0.89(0.86,0.91)	<0.0001
Racquet sport	68/195	0.99(0.78,1.26)	0.95		336/1021	0.81(0.73,0.90)	0.0001
Golf					1210/2983	0.87(0.82,0.92)	<0.0001
Walking for exercise	1210/2983	0.87(0.82,0.92)	<0.0001				

HRs represent mortality risks for those achieving 7.5-<15 MET hrs/wk through a combination of two activities in comparison with those who did not participate in either activity. HRs were adjusted for age, sex, racial/ethnic group, education, smoking status, BMI, alcohol consumption, marriage status, trouble with physical, history of stroke, history of heart attack, angina, or coronary artery disease, history of diabetes, ever been diagnosed with cancer, total MET hours per week from non-leisure time activities, sedentary time, weight training frequency and total MET hours per week from other leisure time activities (excluding the two activities of interest).

Abbreviations: CI=confidence interval; HR=hazard ratio; MET=metabolic equivalent of task; NIH-AARP=National Institutes of Health-AARP.

© 2022 Watts EL et al. *JAMA Network Open.*

eTable 9. Associations of Each Activity With All-Cause, Cardiovascular and Cancer Mortality for NIH-AARP Diet and Health Study Participants (Any vs None)

			All-cause mortality				Cardiovascular mortality				Cancer mortality		
			Deaths	HR (95% CI)	P		Deaths	HR (95% CI)	P		Deaths	HR (95% CI)	P
Running	None		112,325	1 (ref)			36,457	1 (ref)			30,637	1 (ref)	
	Any		5,828	0.89 (0.87, 0.92)	<0.0001		1,843	0.91 (0.87, 0.96)	0.0003		1,729	0.88 (0.84, 0.93)	<0.0001
Cycling	None		91,052	1 (ref)			29,284	1 (ref)			25,099	1 (ref)	
	Any		27,101	0.95 (0.93, 0.96)	<0.0001		9,016	0.96 (0.94, 0.99)	0.002		7,267	0.92 (0.89, 0.94)	<0.0001
Swimming	None		107,366	1 (ref)			34,749	1 (ref)			29,313	1 (ref)	
	Any		10,787	0.94 (0.92, 0.95)	<0.0001		3,551	0.96 (0.93, 1.00)	0.03		3,053	0.95 (0.91, 0.98)	0.003
Aerobic exercise	None		86,023	1 (ref)			27,922	1 (ref)			23,422	1 (ref)	
	Any		32,130	0.94 (0.93, 0.95)	<0.0001		10,378	0.94 (0.92, 0.96)	<0.0001		8,944	0.95 (0.93, 0.98)	0.0009
Racquet sports	None		114,759	1 (ref)			37,274	1 (ref)			31,293	1 (ref)	
	Any		3,394	0.82 (0.79, 0.85)	<0.0001		1,026	0.79 (0.74, 0.84)	<0.0001		1,073	0.89 (0.84, 0.94)	0.0002
Golf	None		102,175	1 (ref)			33,283	1 (ref)			27,418	1 (ref)	
	Any		15,978	0.88 (0.86, 0.89)	<0.0001		5,017	0.84 (0.82, 0.87)	<0.0001		4,948	0.96 (0.93, 0.99)	0.007
Walking for exercise	None		28,506	1 (ref)			9,197	1 (ref)			7,736	1 (ref)	
	Any		89,647	0.94 (0.93, 0.95)	<0.0001		29,103	0.93 (0.90, 0.95)	<0.0001		24,630	0.96 (0.94, 0.99)	0.003
Test for heterogeneity					<0.0001				<0.0001				0.003

HRs represent mortality risks for participation in any amount of physical activity in comparison with who did not participate in the activity.
HRs were adjusted for age, sex, racial/ethnic group, education, smoking status, BMI, alcohol consumption, marriage status, trouble with physical, history of stroke, history of heart attack, angina, or coronary artery disease, history of diabetes, ever been diagnosed with cancer, total MET hours per week from non-leisure time activities, sedentary time, weight training frequency and total MET hours per week from other leisure time activities (excluding the activity of interest).
Heterogeneity between HRs for each activity type and mortality risk was assessed using the $\chi 2$ test.
Abbreviations: CI=confidence interval; HR=hazard ratio; MET=metabolic equivalent of task; NIH-AARP=National Institutes of Health-AARP.

© 2022 Watts EL et al. *JAMA Network Open.*

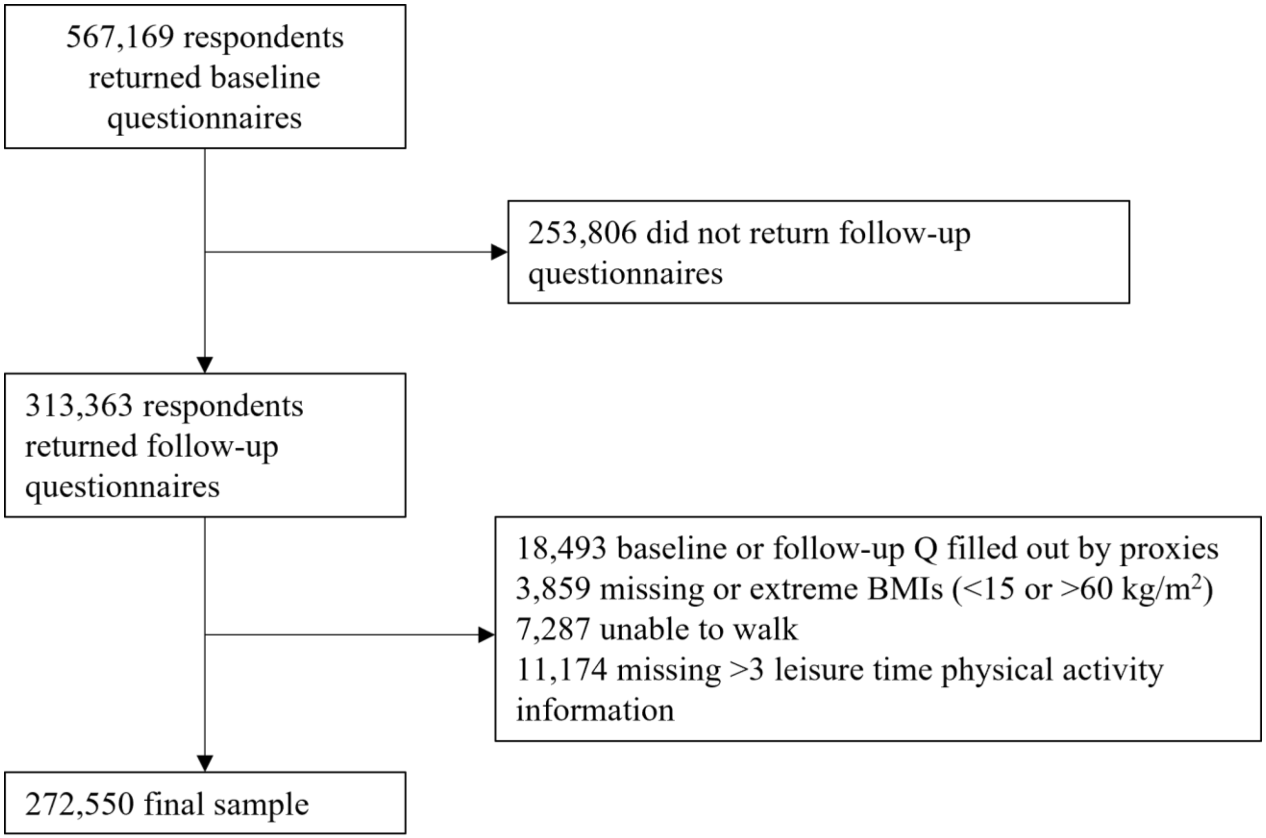

eFigure 1. Participant Inclusion Flow Chart

© 2022 Watts EL et al. *JAMA Network Open.*

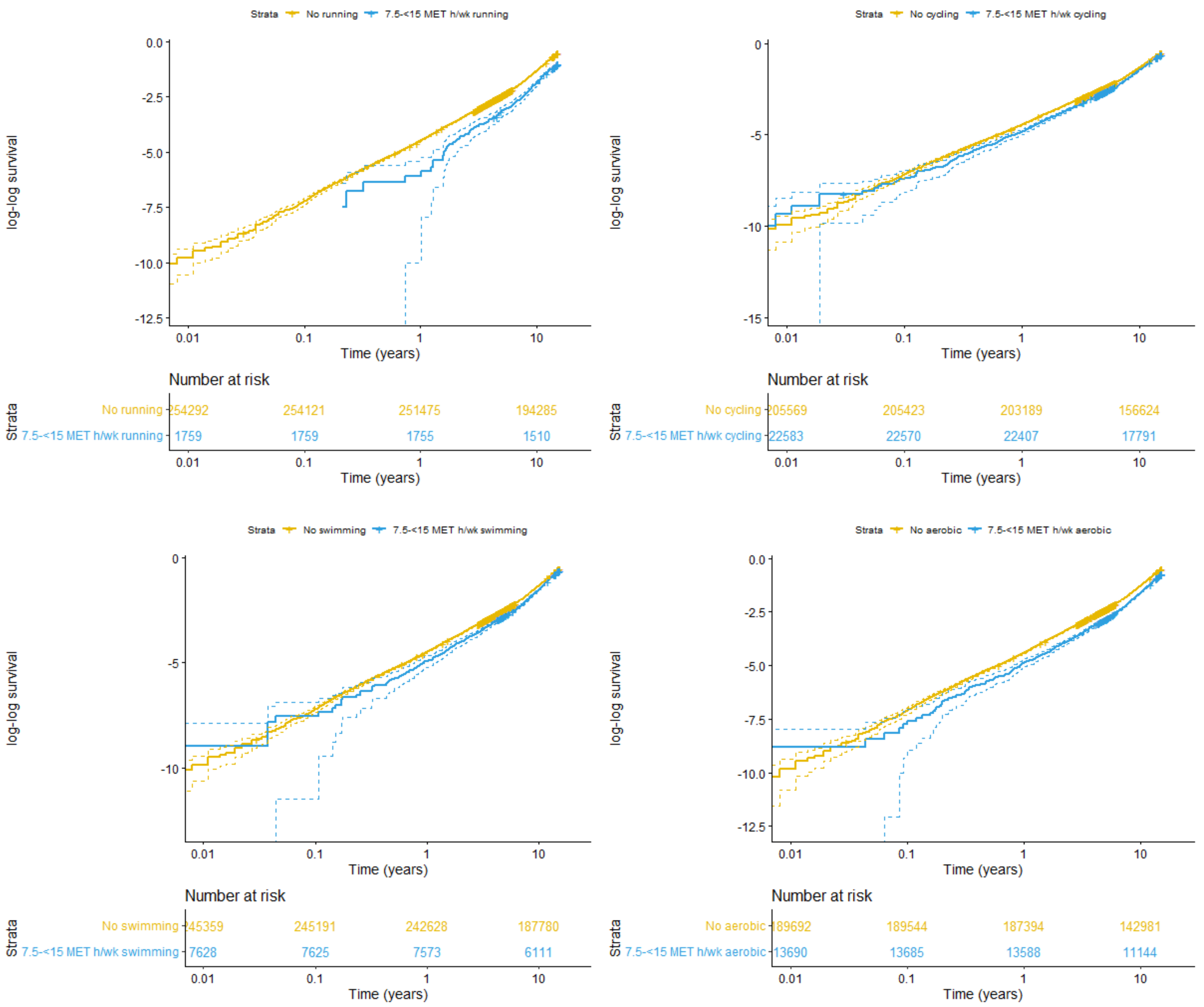

© 2022 Watts EL et al. *JAMA Network Open.*

eFigure 2. Log-Log Plots to Assess Proportional Hazards Assumption With All-Cause Mortality

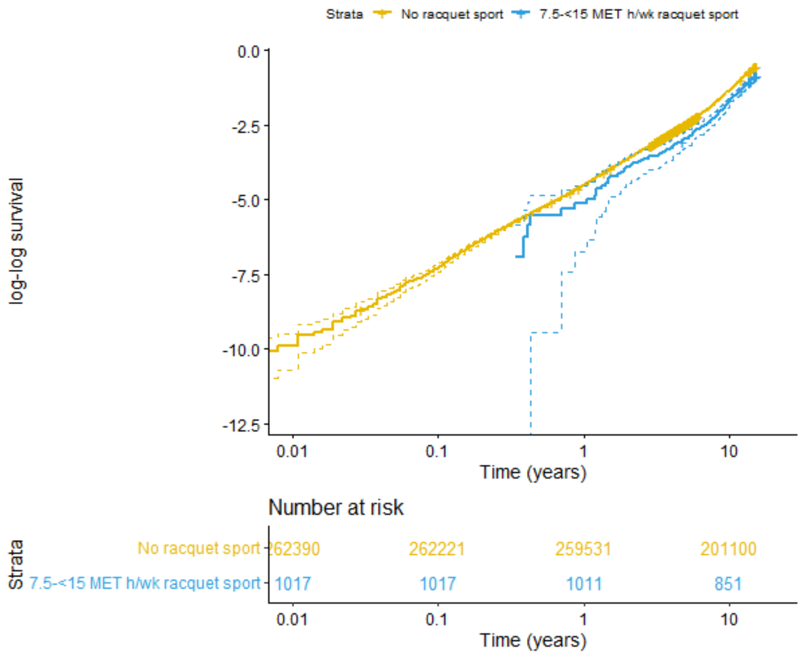

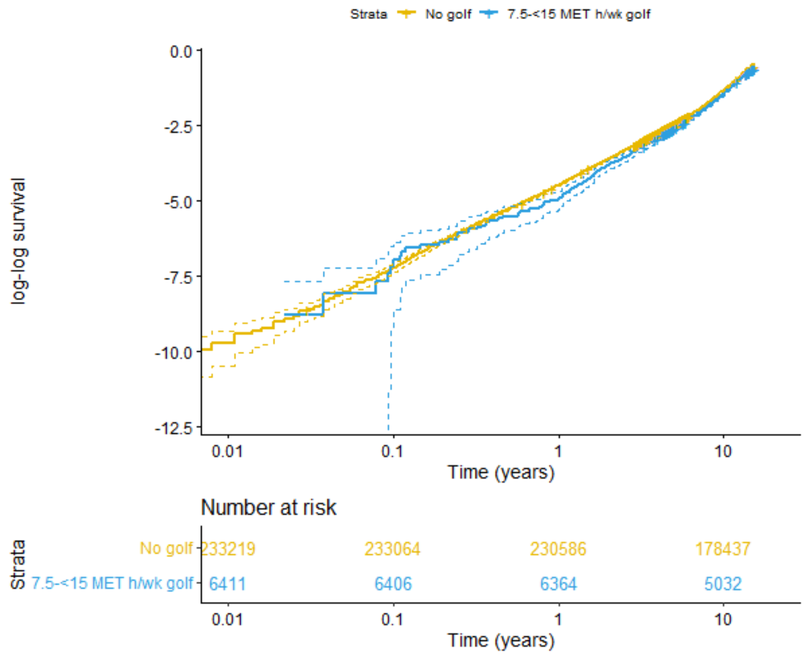

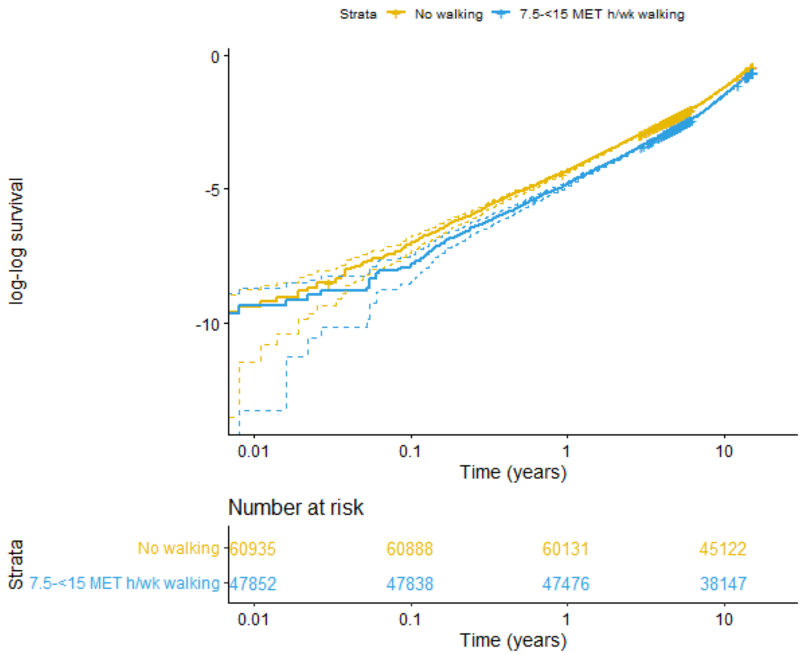

© 2022 Watts EL et al. *JAMA Network Open.*

eFigure 2. Log-Log Plots to Assess Proportional Hazards Assumption With All-Cause Mortality (continued)

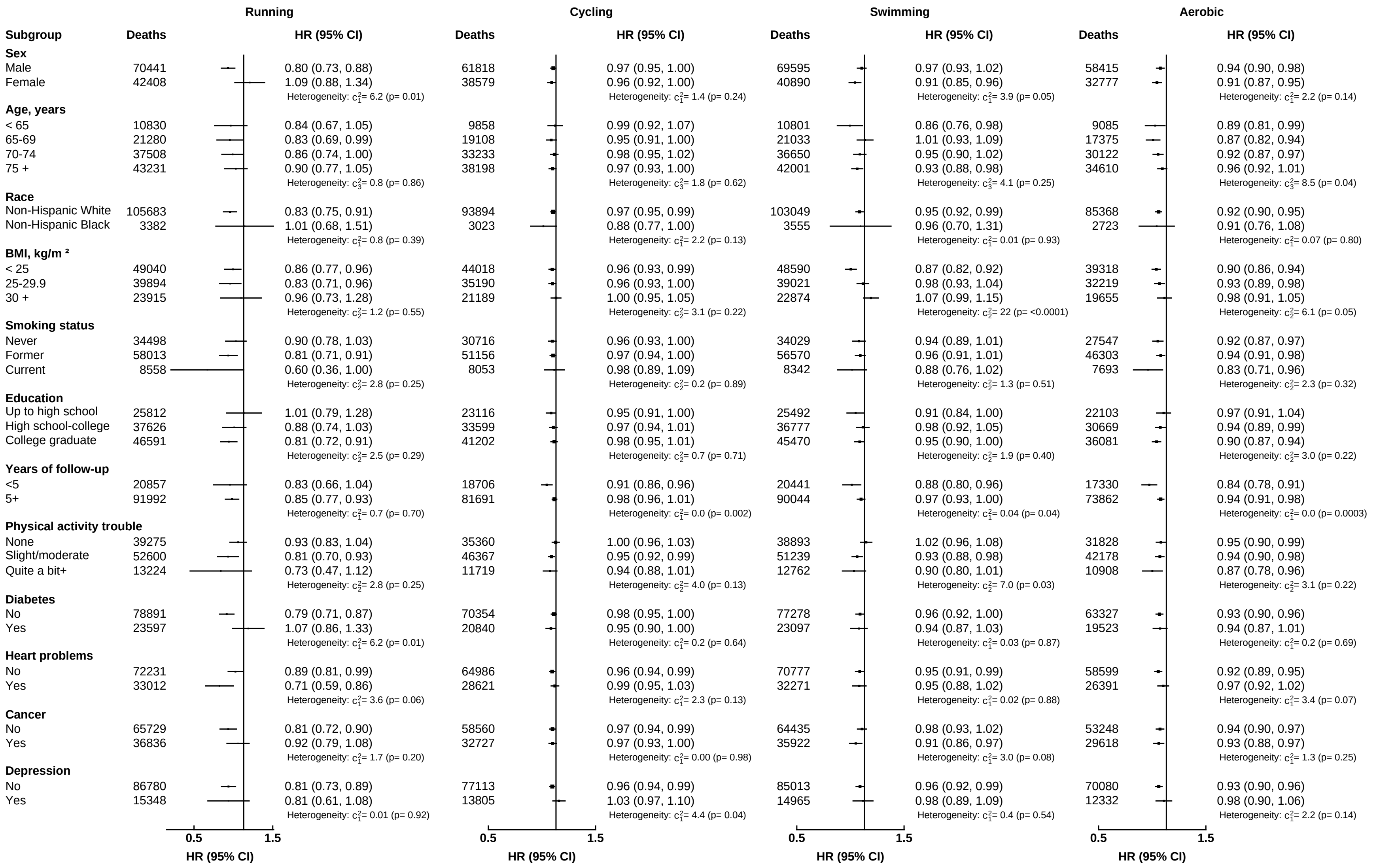

Subgroup	Running Deaths	Running HR (95% CI)	Cycling Deaths	Cycling HR (95% CI)	Swimming Deaths	Swimming HR (95% CI)	Aerobic Deaths	Aerobic HR (95% CI)
**Sex**								
Male	70441	0.80 (0.73, 0.88)	61818	0.97 (0.95, 1.00)	69595	0.97 (0.93, 1.02)	58415	0.94 (0.90, 0.98)
Female	42408	1.09 (0.88, 1.34)	38579	0.96 (0.92, 1.00)	40890	0.91 (0.85, 0.96)	32777	0.91 (0.87, 0.95)
		Heterogeneity: c_1^2= 6.2 (p= 0.01)		Heterogeneity: c_1^2= 1.4 (p= 0.24)		Heterogeneity: c_1^2= 3.9 (p= 0.05)		Heterogeneity: c_1^2= 2.2 (p= 0.14)
**Age, years**								
< 65	10830	0.84 (0.67, 1.05)	9858	0.99 (0.92, 1.07)	10801	0.86 (0.76, 0.98)	9085	0.89 (0.81, 0.99)
65-69	21280	0.83 (0.69, 0.99)	19108	0.95 (0.91, 1.00)	21033	1.01 (0.93, 1.09)	17375	0.87 (0.82, 0.94)
70-74	37508	0.86 (0.74, 1.00)	33233	0.98 (0.95, 1.02)	36650	0.95 (0.90, 1.02)	30122	0.92 (0.87, 0.97)
75 +	43231	0.90 (0.77, 1.05)	38198	0.97 (0.93, 1.00)	42001	0.93 (0.88, 0.98)	34610	0.96 (0.92, 1.01)
		Heterogeneity: c_3^2= 0.8 (p= 0.86)		Heterogeneity: c_3^2= 1.8 (p= 0.62)		Heterogeneity: c_3^2= 4.1 (p= 0.25)		Heterogeneity: c_3^2= 8.5 (p= 0.04)
**Race**								
Non-Hispanic White	105683	0.83 (0.75, 0.91)	93894	0.97 (0.95, 0.99)	103049	0.95 (0.92, 0.99)	85368	0.92 (0.90, 0.95)
Non-Hispanic Black	3382	1.01 (0.68, 1.51)	3023	0.88 (0.77, 1.00)	3555	0.96 (0.70, 1.31)	2723	0.91 (0.76, 1.08)
		Heterogeneity: c_1^2= 0.8 (p= 0.39)		Heterogeneity: c_1^2= 2.2 (p= 0.13)		Heterogeneity: c_1^2= 0.01 (p= 0.93)		Heterogeneity: c_1^2= 0.07 (p= 0.80)
**BMI, kg/m ²**								
< 25	49040	0.86 (0.77, 0.96)	44018	0.96 (0.93, 0.99)	48590	0.87 (0.82, 0.92)	39318	0.90 (0.86, 0.94)
25-29.9	39894	0.83 (0.71, 0.96)	35190	0.96 (0.93, 1.00)	39021	0.98 (0.93, 1.04)	32219	0.93 (0.89, 0.98)
30 +	23915	0.96 (0.73, 1.28)	21189	1.00 (0.95, 1.05)	22874	1.07 (0.99, 1.15)	19655	0.98 (0.91, 1.05)
		Heterogeneity: c_2^2= 1.2 (p= 0.55)		Heterogeneity: c_2^2= 3.1 (p= 0.22)		Heterogeneity: c_2^2= 22 (p= <0.0001)		Heterogeneity: c_2^2= 6.1 (p= 0.05)
**Smoking status**								
Never	34498	0.90 (0.78, 1.03)	30716	0.96 (0.93, 1.00)	34029	0.94 (0.89, 1.01)	27547	0.92 (0.87, 0.97)
Former	58013	0.81 (0.71, 0.91)	51156	0.97 (0.94, 1.00)	56570	0.96 (0.91, 1.01)	46303	0.94 (0.91, 0.98)
Current	8558	0.60 (0.36, 1.00)	8053	0.98 (0.89, 1.09)	8342	0.88 (0.76, 1.02)	7693	0.83 (0.71, 0.96)
		Heterogeneity: c_2^2= 2.8 (p= 0.25)		Heterogeneity: c_2^2= 0.2 (p= 0.89)		Heterogeneity: c_2^2= 1.3 (p= 0.51)		Heterogeneity: c_2^2= 2.3 (p= 0.32)
**Education**								
Up to high school	25812	1.01 (0.79, 1.28)	23116	0.95 (0.91, 1.00)	25492	0.91 (0.84, 1.00)	22103	0.97 (0.91, 1.04)
High school-college	37626	0.88 (0.74, 1.03)	33599	0.97 (0.94, 1.01)	36777	0.98 (0.92, 1.05)	30669	0.94 (0.89, 0.99)
College graduate	46591	0.81 (0.72, 0.91)	41202	0.98 (0.95, 1.01)	45470	0.95 (0.90, 1.00)	36081	0.90 (0.87, 0.94)
		Heterogeneity: c_2^2= 2.5 (p= 0.29)		Heterogeneity: c_2^2= 0.7 (p= 0.71)		Heterogeneity: c_2^2= 1.9 (p= 0.40)		Heterogeneity: c_2^2= 3.0 (p= 0.22)
**Years of follow-up**								
<5	20857	0.83 (0.66, 1.04)	18706	0.91 (0.86, 0.96)	20441	0.88 (0.80, 0.96)	17330	0.84 (0.78, 0.91)
5+	91992	0.85 (0.77, 0.93)	81691	0.98 (0.96, 1.01)	90044	0.97 (0.93, 1.00)	73862	0.94 (0.91, 0.98)
		Heterogeneity: c_1^2= 0.7 (p= 0.70)		Heterogeneity: c_1^2= 0.0 (p= 0.002)		Heterogeneity: c_1^2= 0.04 (p= 0.04)		Heterogeneity: c_1^2= 0.0 (p= 0.0003)
**Physical activity trouble**								
None	39275	0.93 (0.83, 1.04)	35360	1.00 (0.96, 1.03)	38893	1.02 (0.96, 1.08)	31828	0.95 (0.90, 0.99)
Slight/moderate	52600	0.81 (0.70, 0.93)	46367	0.95 (0.92, 0.99)	51239	0.93 (0.88, 0.98)	42178	0.94 (0.90, 0.98)
Quite a bit+	13224	0.73 (0.47, 1.12)	11719	0.94 (0.88, 1.01)	12762	0.90 (0.80, 1.01)	10908	0.87 (0.78, 0.96)
		Heterogeneity: c_2^2= 2.8 (p= 0.25)		Heterogeneity: c_2^2= 4.0 (p= 0.13)		Heterogeneity: c_2^2= 7.0 (p= 0.03)		Heterogeneity: c_2^2= 3.1 (p= 0.22)
**Diabetes**								
No	78891	0.79 (0.71, 0.87)	70354	0.98 (0.95, 1.00)	77278	0.96 (0.92, 1.00)	63327	0.93 (0.90, 0.96)
Yes	23597	1.07 (0.86, 1.33)	20840	0.95 (0.90, 1.00)	23097	0.94 (0.87, 1.03)	19523	0.94 (0.87, 1.01)
		Heterogeneity: c_1^2= 6.2 (p= 0.01)		Heterogeneity: c_1^2= 0.2 (p= 0.64)		Heterogeneity: c_1^2= 0.03 (p= 0.87)		Heterogeneity: c_1^2= 0.2 (p= 0.69)
**Heart problems**								
No	72231	0.89 (0.81, 0.99)	64986	0.96 (0.94, 0.99)	70777	0.95 (0.91, 0.99)	58599	0.92 (0.89, 0.95)
Yes	33012	0.71 (0.59, 0.86)	28621	0.99 (0.95, 1.03)	32271	0.95 (0.88, 1.02)	26391	0.97 (0.92, 1.02)
		Heterogeneity: c_1^2= 3.6 (p= 0.06)		Heterogeneity: c_1^2= 2.3 (p= 0.13)		Heterogeneity: c_1^2= 0.02 (p= 0.88)		Heterogeneity: c_1^2= 3.4 (p= 0.07)
**Cancer**								
No	65729	0.81 (0.72, 0.90)	58560	0.97 (0.94, 0.99)	64435	0.98 (0.93, 1.02)	53248	0.94 (0.90, 0.97)
Yes	36836	0.92 (0.79, 1.08)	32727	0.97 (0.93, 1.00)	35922	0.91 (0.86, 0.97)	29618	0.93 (0.88, 0.97)
		Heterogeneity: c_1^2= 1.7 (p= 0.20)		Heterogeneity: c_1^2= 0.00 (p= 0.98)		Heterogeneity: c_1^2= 3.0 (p= 0.08)		Heterogeneity: c_1^2= 1.3 (p= 0.25)
**Depression**								
No	86780	0.81 (0.73, 0.89)	77113	0.96 (0.94, 0.99)	85013	0.96 (0.92, 0.99)	70080	0.93 (0.90, 0.96)
Yes	15348	0.81 (0.61, 1.08)	13805	1.03 (0.97, 1.10)	14965	0.98 (0.89, 1.09)	12332	0.98 (0.90, 1.06)
		Heterogeneity: c_1^2= 0.01 (p= 0.92)		Heterogeneity: c_1^2= 4.4 (p= 0.04)		Heterogeneity: c_1^2= 0.4 (p= 0.54)		Heterogeneity: c_1^2= 2.2 (p= 0.14)

eFigure 3. Associations of Activity Types With All-Cause Mortality by Subgroups

HRs for the associations of meeting the recommended physical activity levels through different leisure time physical activities (7.5-<15 MET hrs/wk vs 0) with all-cause mortality, stratified by subgroups. HRs were adjusted for age, sex, racial/ethnic group, education, smoking status, BMI, alcohol consumption, marriage status, trouble with physical, history of stroke, history of heart attack, angina, or coronary artery disease, history of diabetes, ever been diagnosed with cancer, total MET hours per week from non-leisure time activities, sedentary time, weight training frequency and total MET hours per week from other leisure time activities (excluding the activity of interest). Heterogeneity in the associations for follow-up time was examined using in two different subgroups defined by follow-up period, using a $\chi 2$ for heterogeneity. Heterogeneity in the associations for the non-case dependent subgroups was assessed using a $\chi 2$ interaction term. The boxes represent the HRs, and vertical lines represent 95% CIs.

Abbreviations: CI=confidence interval; HR=hazard ratio; MET= metabolic equivalent of task.

© 2022 Watts EL et al. *JAMA Network Open.*

Subgroup	Racquet sports Deaths	Racquet sports HR (95% CI)	Golf Deaths	Golf HR (95% CI)	Walking Deaths	Walking HR (95% CI)
**Sex**						
Male	72127	0.85 (0.76, 0.96)	62742	0.95 (0.91, 0.99)	30137	0.93 (0.90, 0.95)
Female	42979	0.79 (0.62, 1.01)	42091	0.86 (0.79, 0.95)	17694	0.88 (0.86, 0.91)
		Heterogeneity: c^2_1= 0.4 (p= 0.55)		Heterogeneity: c^2_1= 3.9 (p= 0.05)		Heterogeneity: c^2_1= 8.6 (p= 0.004)
**Age, years**						
< 65	11313	0.78 (0.54, 1.12)	10387	0.95 (0.83, 1.07)	4840	0.98 (0.92, 1.04)
65-69	21950	0.98 (0.78, 1.22)	19971	0.89 (0.81, 0.97)	9226	0.92 (0.88, 0.96)
70-74	38146	0.88 (0.74, 1.05)	34770	0.92 (0.86, 0.98)	15702	0.89 (0.86, 0.92)
75 +	43697	0.74 (0.61, 0.89)	39705	0.97 (0.91, 1.03)	18063	0.90 (0.87, 0.93)
		Heterogeneity: c^2_3= 4.0 (p= 0.26)		Heterogeneity: c^2_3= 3.0 (p= 0.39)		Heterogeneity: c^2_3= 6.3 (p= 0.10)
**Race**						
Non-Hispanic white	107495	0.83 (0.75, 0.93)	97533	0.94 (0.90, 0.97)	45018	0.91 (0.89, 0.93)
Non-Hispanic black	3603	1.31 (0.68, 2.53)	3522	1.03 (0.75, 1.40)	1276	0.99 (0.88, 1.12)
		Heterogeneity: c^2_1= 1.9 (p= 0.17)		Heterogeneity: c^2_1= 0.17 (p= 0.68)		Heterogeneity: c^2_1= 2.1 (p= 0.14)
**BMI, kg/m ²**						
< 25	50169	0.80 (0.69, 0.92)	46298	0.95 (0.90, 1.01)	20972	0.91 (0.89, 0.94)
25-29.9	40694	0.93 (0.78, 1.10)	36112	0.93 (0.87, 0.99)	16586	0.92 (0.89, 0.95)
30 +	24243	0.82 (0.57, 1.17)	22423	0.90 (0.81, 0.99)	10273	0.90 (0.87, 0.94)
		Heterogeneity: c^2_2= 2.0 (p= 0.36)		Heterogeneity: c^2_2= 0.9 (p= 0.65)		Heterogeneity: c^2_2= 0.4 (p= 0.81)
**Smoking status**						
Never	35324	0.76 (0.63, 0.91)	33393	0.99 (0.92, 1.07)	14153	0.94 (0.91, 0.97)
Former	59015	0.86 (0.74, 0.99)	52437	0.91 (0.87, 0.96)	24574	0.90 (0.88, 0.92)
Current	8719	0.95 (0.55, 1.64)	7996	0.84 (0.71, 0.99)	4126	0.90 (0.84, 0.97)
		Heterogeneity: c^2_2= 1.2 (p= 0.55)		Heterogeneity: c^2_2= 5.0 (p= 0.08)		Heterogeneity: c^2_2= 3.4 (p= 0.19)
**Education**						
Up to high school	26353	0.76 (0.55, 1.06)	24809	0.88 (0.80, 0.97)	10925	0.89 (0.86, 0.93)
High school-college	38478	0.94 (0.74, 1.18)	35056	0.87 (0.82, 0.94)	15985	0.91 (0.88, 0.94)
College graduate	47383	0.83 (0.73, 0.94)	42266	0.99 (0.94, 1.05)	19785	0.92 (0.89, 0.94)
		Heterogeneity: c^2_2= 1.1 (p= 0.58)		Heterogeneity: c^2_2= 8.7 (p= 0.01)		Heterogeneity: c^2_2= 1.0 (p= 0.61)
**Years of follow-up**						
<5	21186	0.81 (0.62, 1.07)	19550	0.91 (0.83, 1.00)	8830	0.84 (0.80, 0.88)
5+	93920	0.84 (0.75, 0.95)	85283	0.94 (0.90, 0.98)	39001	0.93 (0.91, 0.95)
		Heterogeneity: c^2_1= 0.7 (p= 0.73)		Heterogeneity: c^2_1= 0.4 (p= 0.45)		Heterogeneity: c^2_1= 0.00 (p= <0.0001)
**Physical activity trouble**						
None	40276	0.76 (0.64, 0.90)	35983	0.94 (0.88, 0.99)	16564	0.94 (0.91, 0.97)
Slight/moderate	53596	0.91 (0.78, 1.06)	48994	0.91 (0.86, 0.96)	21788	0.91 (0.89, 0.94)
Quite a bit+	13324	0.86 (0.51, 1.42)	12650	0.94 (0.81, 1.10)	5942	0.87 (0.82, 0.93)
		Heterogeneity: c^2_2= 2.3 (p= 0.31)		Heterogeneity: c^2_2= 0.7 (p= 0.71)		Heterogeneity: c^2_2= 7.3 (p= 0.03)
**Diabetes**						
No	80398	0.84 (0.75, 0.95)	73034	0.94 (0.90, 0.99)	33802	0.92 (0.90, 0.95)
Yes	24143	0.72 (0.52, 0.99)	22272	0.94 (0.86, 1.03)	9675	0.89 (0.85, 0.92)
		Heterogeneity: c^2_1= 1.0 (p= 0.33)		Heterogeneity: c^2_1= 0.01 (p= 0.94)		Heterogeneity: c^2_1= 3.2 (p= 0.07)
**Heart problems**						
No	73814	0.81 (0.71, 0.92)	67657	0.95 (0.90, 0.99)	31021	0.93 (0.91, 0.95)
Yes	33541	0.87 (0.70, 1.07)	30092	0.91 (0.84, 0.97)	13607	0.88 (0.85, 0.91)
		Heterogeneity: c^2_1= 0.4 (p= 0.52)		Heterogeneity: c^2_1= 1.1 (p= 0.29)		Heterogeneity: c^2_1= 5.5 (p= 0.02)
**Cancer**						
No	67243	0.81 (0.70, 0.93)	61430	0.88 (0.84, 0.93)	27727	0.91 (0.89, 0.93)
Yes	37328	0.89 (0.75, 1.06)	33797	0.98 (0.92, 1.05)	15570	0.92 (0.89, 0.95)
		Heterogeneity: c^2_1= 0.7 (p= 0.41)		Heterogeneity: c^2_1= 6.1 (p= 0.01)		Heterogeneity: c^2_1= 0.06 (p= 0.80)
**Depression**						
No	88507	0.81 (0.72, 0.91)	79949	0.93 (0.89, 0.97)	36912	0.92 (0.90, 0.94)
Yes	15688	1.21 (0.88, 1.66)	14943	0.94 (0.82, 1.07)	6433	0.89 (0.84, 0.93)
		Heterogeneity: c^2_1= 5.9 (p= 0.01)		Heterogeneity: c^2_1= 0.03 (p= 0.87)		Heterogeneity: c^2_1= 2.4 (p= 0.12)

HR (95% CI) axis: 0.5 – 1.5 (each panel)

eFigure 3. Associations of Activity Types With All-Cause Mortality by Subgroups (continued)
HRs for the associations of meeting the recommended physical activity levels through different leisure time physical activities with all-cause mortality (7.5-<15 MET hrs/wk vs 0), stratified by subgroups. HRs were adjusted for age, sex, racial/ethnic group, education, smoking status, BMI, alcohol consumption, marriage status, trouble with physical, history of stroke, history of heart attack, angina, or coronary artery disease, history of diabetes, ever been diagnosed with cancer, total MET hours per week from non-leisure time activities, sedentary time, weight training frequency and total MET hours per week from other leisure time activities (excluding the activity of interest). Heterogeneity in the associations for follow-up time was examined using in two different subgroups defined by follow-up period, using a $\chi2$ for heterogeneity. Heterogeneity in the associations for the non-case dependent subgroups was assessed using a $\chi2$ interaction term. The boxes represent the HRs, and vertical lines represent 95% CIs.
Abbreviations: CI=confidence interval; HR=hazard ratio; MET= metabolic equivalent of task.

© 2022 Watts EL et al. *JAMA Network Open.*

eReferences.

1 Ainsworth BE, Haskell WL, Herrmann SD et al. 2011 Compendium of Physical Activities: a second update of codes and MET values. Med Sci Sports Exerc 2011; 43 (8): 1575-1581.

2 Schneider KL, Andrews C, Hovey KM et al. Change in physical activity after a diabetes diagnosis: opportunity for intervention. Med Sci Sports Exerc 2014; 46 (1): 84-91.

3 Saunders DH, Greig CA, Mead GE. Physical activity and exercise after stroke: review of multiple meaningful benefits. Stroke 2014; 45 (12): 3742-3747.

4 Irwin ML, Crumley D, McTiernan A et al. Physical activity levels before and after a diagnosis of breast carcinoma: the Health, Eating, Activity, and Lifestyle (HEAL) study. Cancer 2003; 97 (7): 1746-1757.

5 Mathur MB, Ding P, Riddell CA et al. Web Site and R Package for Computing E-values. Epidemiology (Cambridge, Mass) 2018; 29 (5): e45-e47.

6 VanderWeele TJ, Ding P. Sensitivity Analysis in Observational Research: Introducing the E-Value. Ann Intern Med 2017; 167 (4): 268-274.

© 2022 Watts EL et al. *JAMA Network Open*.
